# Association of the C-reactive protein–triglyceride–glucose index with liver disease risk: findings from a nationwide Chinese cohort

**DOI:** 10.1186/s12876-025-04588-2

**Published:** 2026-01-03

**Authors:** Jinpeng Zheng, Dongwei Xie, Yuanyuan Nong, Kui Jia, Dingran Sha

**Affiliations:** 1 Department of Hepatobiliary Surgery, The First Affiliated Hospital of Guangxi Medical University, Nanning, Guangxi China; 2 Department of Ultrasound, The First Affiliated Hospital of Guangxi Medical University, Nanning, Guangxi China; 3 Department of Internal Medicine, Guangxi Medical University Cancer Hospital, Nanning, Guangxi China; 4 Department of Nursing, The First Affiliated Hospital of Guangxi Medical University, Nanning, Guangxi China; 5 Department of Urology, The First Affiliated Hospital of Guangxi Medical University, Shuangyong Road NO.6, Nanning, Guangxi 530021 China

**Keywords:** Liver disease, CTI index, Insulin resistance, Inflammation, Sex differences, CHARLS

## Abstract

**Background:**

Both insulin resistance and low-grade inflammation contribute to liver disease development, yet their combined predictive role remains unclear at the population level.

**Methods:**

In this prospective nationwide cohort study, we analyzed 9,302 participants aged ≥ 45 years from the China Health and Retirement Longitudinal Study who were free of liver disease at baseline. Additionally, CTI was calculated as 0.412 × ln (C-reactive protein [CRP, mg/L]) + ln((triglycerides [TG, mg/dL] × fasting plasma glucose [FPG, mg/dL])/2). Incident self-reported, physician-diagnosed liver disease was identified during follow-up interviews. Associations between CTI and liver disease were assessed using Cox regression, Kaplan–Meier survival curves, and restricted cubic splines analyses. Subgroup and mediation analyses were conducted for low-density and high-density lipoprotein cholesterol.

**Results:**

Over 81,229 person-years of follow-up, higher CTI was independently associated with an increased risk of incident self-reported, physician-diagnosed liver disease, with risk increasing progressively across CTI quartiles. This positive association was consistent in men and women, with approximately linear dose–response relationships in both sexes and no meaningful sex differences. Mediation through low- and high-density lipoprotein cholesterol was negligible. Findings remain robust in multiple sensitivity analyses, including exclusion of participants who reported high-risk alcohol consumption.

**Conclusion:**

Elevated CTI is a simple, low-cost biomarker that may help identify individuals at increased risk of liver disease, particularly in settings where comprehensive liver function panels are unavailable. Rather than replacing guideline-recommended fibrosis scores, CTI may serve as a complementary early risk indicator to support population-level risk stratification and inform targeted prevention and follow-up efforts.

**Supplementary Information:**

The online version contains supplementary material available at 10.1186/s12876-025-04588-2.

## Introduction

Liver disease (LD) encompasses a progressive spectrum from steatosis and inflammation to fibrosis, cirrhosis, liver failure, and hepatocellular carcinoma and constitutes a major cause of global morbidity and mortality. Globally, LD accounts for approximately 4% of all deaths, largely attributable to cirrhosis and liver cancer, with the burden particularly high in aging populations [1, 2]. In China, more than one-fifth of adults are affected [3]. Recent multicenter data indicated a high prevalence of hepatic steatosis and fibrosis among older adults, underscoring the need for scalable tools to identify high-risk groups [4]. 

Consistent with recent multi-society consensus statements, we used “steatotic liver disease” as the umbrella term and referred to the metabolic dysfunction–associated subtype as “metabolic dysfunction-associated steatotic liver disease” (MASLD), and acknowledged that earlier literature relied on the historical term “non-alcoholic fatty liver disease” [5, 6]. MASLD is strongly linked to metabolic syndrome components, including type 2 diabetes, hypertension, dyslipidemia, and obesity, and can advance to fibrosis and end-stage liver disease [7]. Therefore, there is growing interest in simple, non-invasive markers that capture upstream metabolic and inflammatory risk before clinically overt LD emerges.

Insulin resistance and chronic low-grade inflammation are central mechanisms in the development and progression of metabolic LD [8]. The triglyceride–glucose (TyG) index, a commonly used surrogate marker of insulin resistance, has been associated with adverse outcomes in MASLD [9—12]. C-reactive protein (CRP), an established marker of systemic inflammation, predicts progression to cirrhosis and hepatic decompensation [13]. However, most previous studies have evaluated insulin resistance–related and inflammation-related markers separately, potentially underestimating the combined impact of metabolic and inflammatory stress on LD risk at the population level.

The C-reactive protein–triglyceride–glucose (CTI) index integrates CRP with triglycerides (TG) and fasting plasma glucose (FPG) to jointly capture inflammatory and insulin resistance pathways and has demonstrated predictive value for cardiovascular disease, stroke, and cancer outcomes [14–17] . Whether CTI is associated with future LD risk in the general population, and whether this association varies by sex, remains unclear. Therefore, we hypothesized that higher CTI, indicating combined insulin resistance and low-grade inflammation, would be independently associated with an increased risk of incident self-reported, physician-diagnosed LD in middle-aged and older adults, and that sex-specific patterns might exist. Using the nationally representative China Health and Retirement Longitudinal Study (CHARLS), we aimed to evaluate the CTI–LD association, characterize its dose–response relationship, and investigate sex-specific and lipid-related mechanisms.

## Methods

### Study design and population

This study used nationally representative data from CHARLS, a population-based longitudinal survey employing multistage, probability-proportionate-to-size sampling methodology. The baseline survey (Wave 1) began in 2011 and enrolled 17,708 middle-aged and older adults from 450 villages across 150 counties in 28 provinces. Standardized instruments were utilized to collect demographic and health data. Participants were followed up every 2–3 years, and no additional surveys were conducted for the current study. Data from all five available waves (2011, 2013, 2015, 2018, and 2020) were included. Methodological details for CHARLS have been published previously. Institutional ethical approval was granted by the Peking University Institutional Review Board, and all participants provided written informed consent in accordance with the Declaration of Helsinki.

**Fig. 1:**
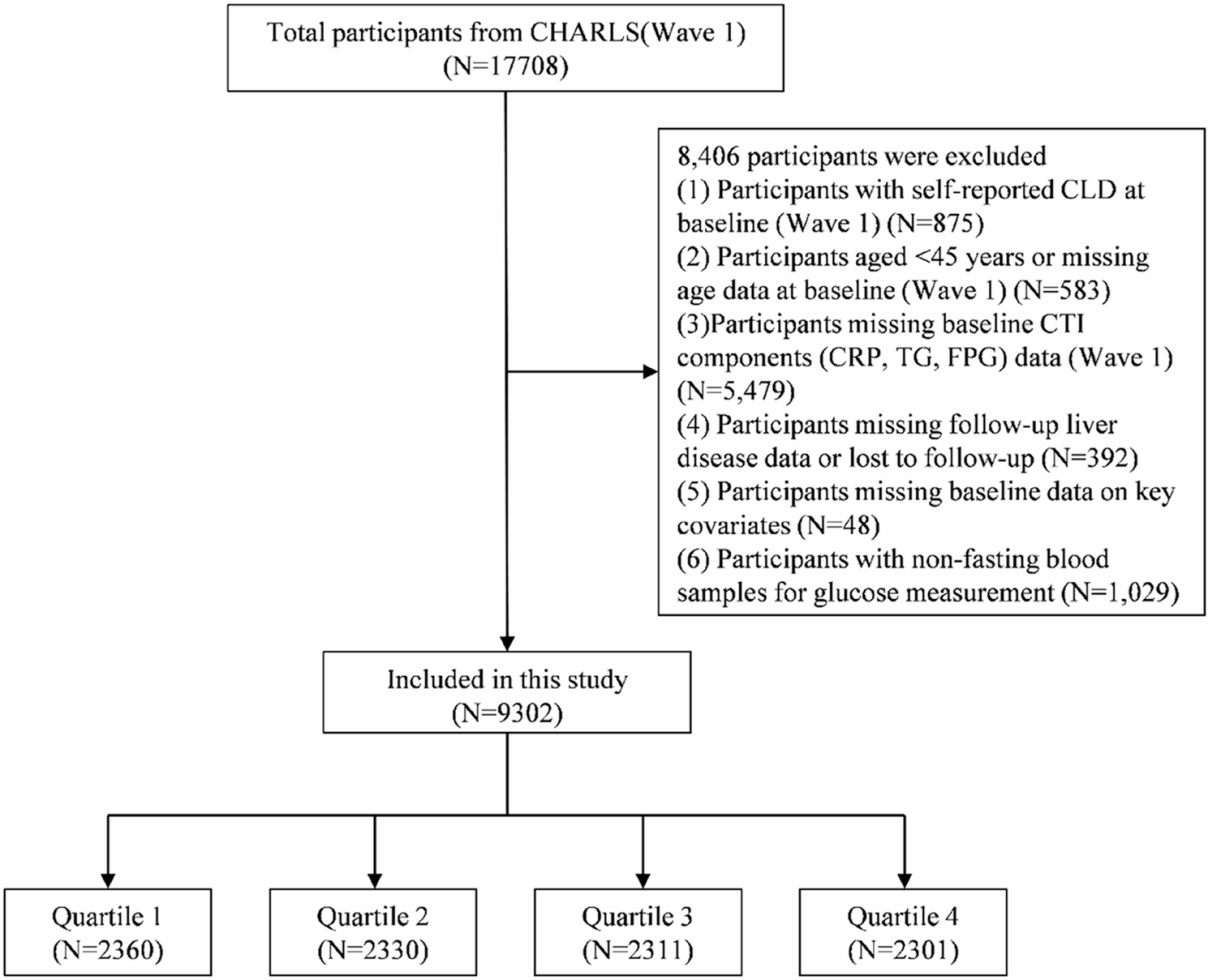
Participant selection flow diagram

Figure 1 outlines participant selection. Of the 17,708 individuals included in Wave 1, considered the baseline cohort, we excluded 875 participants with self-reported LD at baseline; 583 participants with missing age data or age < 45 years; 5,479 participants lacking baseline CTI components (CRP, TG, and FPG); 392 participants without follow-up LD data or lost to follow-up; 48 participants with missing baseline data on key covariates; 1,029 participants with non-fasting blood glucose samples. The final analytical cohort comprised 9,302 eligible participants; 8,406 individuals were excluded from the final analysis.

### Exposure: CTI calculation

CTI was calculated using the following formula as proposed by Ruan et al. [14]:

CTI = 0.412 × Ln(CRP [mg/L]) + Ln((TG [mg/dL] × FPG [mg/dL])/2). In CHARLS, fasting TG and FPG are reported in mmol/L, whereas high-sensitivity CRP is reported in mg/L. Therefore, before computing CTI, TG and FPG were converted to mg/dL using TG (mg/dL) = TG (mmol/L) × 88.57 and FPG (mg/dL) = FPG (mmol/L) × 18.00; CRP was used directly in mg/L as reported.

### Outcome: assessment of LD

The primary outcome was incident self-reported physician-diagnosed LD, defined as a “yes” response to either of the following questions: “Have you been diagnosed with LD by a doctor?” or “Have you taken any medication to treat LD?” The time to LD onset was defined as the interval between the baseline interview and the follow-up wave at which LD was first reported. This broad endpoint reflects participants’ self-report of a prior physician diagnosis or treatment for “liver disease” and does not allow reliable etiologic subtyping (MASLD versus viral, alcohol-related, autoimmune, or drug-induced LD).

### Covariates

Baseline data were collected by trained interviewers utilizing structured questionnaires. (1) Demographics and lifestyle: Information included sex, age, residence, education, marital status, smoking status, alcohol consumption, and sleep duration. For alcohol, although CHARLS does not capture quantitative intake, current drinking behavior is recorded as a nine-level frequency variable. For the present analyses, this variable was recorded into four ordered categories to approximate alcohol consumption: none; occasional drinking (combining “less than once a month,” “once a month,” and “2–3 days a month”); weekly drinking (combining “once a week” and “2–3 days a week”); and near-daily or daily drinking (combining “4–6 days a week,” “daily,” “twice a day,” and “more than twice a day”). This recoded alcohol frequency variable was included as a covariate in multivariable models. (2) Anthropometric measures: Height, weight, body mass index (BMI), waist circumference, systolic blood pressure (SBP), and diastolic blood pressure (DBP) were measured according to standardized protocols. (3) Medical history and medication use: Participants reported the presence of hypertension, heart disease, diabetes, and dyslipidemia, and medication use for these conditions. (4) Laboratory data: Collected biomarkers included FPG, total cholesterol (TC), TG, high-density lipoprotein cholesterol (HDL-C), low-density lipoprotein cholesterol (LDL-C), HbA1c, serum creatinine (Scr), uric acid (UA), and blood urea nitrogen (BUN).

Hypertension was defined by any of the following criteria: (1) SBP ≥ 140 mmHg, (2) DBP ≥ 90 mmHg, (3) self-reported physician diagnosis of hypertension, or (4) current use of antihypertensive medication. Diabetes was defined as (1) FPG ≥ 126 mg/dL, (2) HbA1c ≥ 6.5%, (3) use of glucose-lowering medication, or (4) self-reported physician diagnosis. Dyslipidemia was defined as meeting any of the following: TG ≥ 150 mg/dL, TC ≥ 240 mg/dL, HDL-C < 40 mg/dL, LDL-C ≥ 160 mg/dL, current use of lipid-lowering medication, or self-reported physician diagnosis. Heart disease was defined as current use of cardiac medications or a self-reported physician diagnosis of heart disease.

### Statistical analysis

Baseline characteristics were summarized as mean ± standard deviation or median (interquartile range) for continuous variables and as count (percentage) for categorical variables. Between-group differences were evaluated using one-way analysis of variance (ANOVA) or Kruskal–Wallis tests for continuous variables and χ² tests for categorical variables. Person-time was accrued from baseline to the first incident self-reported physician-diagnosed LD or last follow-up, and incidence rates were reported per 1,000 person-years with 95% confidence intervals (CIs).

The CTI index was modelled both as a continuous exposure (per 1-unit increase) and in quartiles (Q1–Q4, with Q1 as the reference). Cox proportional hazards models were utilized to estimate hazard ratios (HRs) and 95% CIs under three prespecified adjustment schemes. Linear trend across CTI quartiles was tested by entering quartile medians as continuous variables. Standardized absolute risks of incident LD at 5 and 7 years by CTI quartile were derived by averaging predicted survival over the baseline covariate distribution (absolute risk = 1 − S(t)), and standardized absolute risk differences were calculated for Q4 versus Q1.

Dose–response relationships between continuous CTI and LD were assessed using restricted cubic splines. Cumulative incidence across CTI categories was illustrated with Kaplan–Meier curves and compared using log-rank tests. Prespecified subgroup and sex-stratified analyses were performed to investigate effect heterogeneity, with multiplicative interaction terms included in Cox models; all subgroup findings (including *P* for interaction) were interpreted as exploratory. Nonlinearity was evaluated using primary four-knot spline models (knots at the 5th, 35th, 65th, and 95th percentiles of CTI) and in sex-stratified sensitivity analyses using three-knot models with common knots at the 10th, 50th, and 90th percentiles. Overall and nonlinear associations were tested using Wald χ² and likelihood-ratio tests.

To investigate the role of blood lipids, a directed acyclic graph was specified in which CTI affects incident LD directly and indirectly through LDL-C and HDL-C, with shared sociodemographic, lifestyle, and clinical factors treated as confounders. Within this framework, a “total-effect” Cox model was fitted without LDL-C/HDL-C, and a “direct-effect” model was additionally adjusted for these lipids. Single- and parallel-mediator causal mediation analyses were conducted using the mediation and lavaan packages. In mediation models, CTI was treated as a continuous exposure, incident LD as a binary outcome, and LDL-C or HDL-C (standardized to z-scores) as mediators; models were adjusted for the same confounders as the Cox models. The average causal mediation effect, average direct effect, total effect, and proportion mediated were estimated using the interquartile contrast of CTI (75th versus 25th percentile) as the exposure contrast.

The proportional hazards assumption was evaluated utilizing Schoenfeld residuals. Missing covariate data were addressed using multiple imputation by chained equations (m = 30; 20 iterations) with variable-appropriate imputation models, and complete-case analyses were conducted as a robustness check. Additional sensitivity analyses included models with alternative time scales, competing-risk approaches, inverse-probability-of-censoring weighting, mid-interval event time assignment, exclusion of participants with marked inflammation or early events, restriction to participants with repeated CTI measurements, exclusion of high-frequency drinkers, and use of a more stringent LD endpoint requiring physician diagnosis and LD medication, with medicated versus non-medicated events compared descriptively. We additionally conducted an E-value analysis to quantify the minimum strength of unmeasured confounding that would be required to fully explain away the observed CTI–LD association. Supplementary Methods and Supplementary Tables present details and numerical results for all sensitivity analyses. All analyses were performed in R (R Foundation for Statistical Computing), and two-sided *p*-values < 0.05 were considered statistically significant.

## Results

### Population characteristics

The final analytical cohort comprised 9,302 participants following the application of all eligibility criteria. Supplementary Table S1 presents variable-level missingness. During 81,229 person-years of follow-up, 734 participants developed an incident of self-reported physician-diagnosed LD, resulting in an overall incidence rate of 9.04 per 1,000 person-years (95% CI: 8.39–9.69). Multiple imputation (MICE, m = 30) and complete-case analyses (7,414 participants; 579 events; 64,772 person-years) produced highly comparable hazard ratio estimates for CTI and other covariates (Supplementary Tables S2–3, Figure S1).

At baseline, the median interquartile range (IQR) of CRP was 1.04 mg/L (IQR as illustrated in Table 1), and 712 participants (7.7%) exhibited CRP levels > 10 mg/L. Participants were categorized into CTI quartiles: Q1 (≤ 8.16), Q2 (8.16–8.67), Q3 (8.67–9.25), and Q4 (> 9.25). Table 1 presents baseline characteristics across CTI quartiles. Compared with individuals in the lowest quartile (Q1), those in the highest quartile (Q4) were predominantly female, more frequently resided in urban areas, and exhibited a higher prevalence of hypertension, cardiovascular disease, and dyslipidemia. Higher CTI quartiles correlated with adverse metabolic and inflammatory profiles, including higher BMI, waist circumference, FPG, HbA1c, TG, TC, Scr, SUA, BUN, CRP, and blood pressure, and lower HDL-C levels. The incidence rates of LD increased progressively across CTI quartiles (Table S4).

**Table 1:** Baseline demographic, clinical, and laboratory characteristics of participants overall and by CTI quartiles

Variable	Overall	Q1	Q2	Q3	Q4	Test_of_significance
No. of subjects	9302	2360	2330	2311	2301	
Sex						χ²=24.99, *p* < 0.001
Female	4957 (53.3%)	1172 (49.7%)	1217 (52.2%)	1271 (55.0%)	1297 (56.4%)	
Male	4345 (46.7%)	1188 (50.3%)	1113 (47.8%)	1040 (45.0%)	1004 (43.6%)	
Age, year	58 [52–65]	57 [50–64]	58 [52–66]	59 [53–65]	59 [52–65]	χ²=57.96, *p* < 0.001
Height, m	1.577 [1.5–1.6]	1.578 [1.52–1.64]	1.579 [1.52–1.64]	1.573 [1.51–1.64]	1.58 [1.52–1.646]	χ²=7.88, *p* = 0.049
Weight, kg	58.1 [51-66.4]	54.9 [48.7–61.4]	56.55 [50-64.2]	59.4 [51.9–67.6]	63.1 [54.9–71.7]	χ²=663.58, *p* < 0.001
BMI, kg/㎡	23.23 [20.9-25.89]	21.98 [20.0–24.0]	22.69 [20-25.13]	23.82 [21-26.46]	24.82 [22-27.63]	χ²=761.13, *p* < 0.001
WC, cm	85 [78-92.2]	80 [75-86.12]	83 [77–90]	87 [80-93.45]	90.4 [83.4–98]	χ²=1179.2, *p* < 0.001
Sleeping time, h	6 [5–8]	7 [5–8]	6 [5–8]	6 [5–8]	6.5 [5–8]	χ²=2.10, *p* = 0.552
FPG, mg/dl	102.2 [94.5-112.7]	96.48 [90.1–103]	100.4 [94-107.4]	103.3 [96-112.3]	113.8 [102-137.9]	χ²=1701.1, *p* < 0.001
HbA1c,%	5.1 [4.9–5.4]	5 [4.8–5.3]	5.1 [4.9–5.4]	5.2 [4.9–5.4]	5.3 [5-5.8]	χ²=528.15, *p* < 0.001
TC, mg/dl	191.4 [168-216.5]	181.7 [160-204.1]	189 [167-211.9]	194.1 [170.9–218]	203.4 [177-233.5]	χ²=415.78, *p* < 0.001
TG, mg/dl	105.3 [75-153.1]	66.38 [54-80.54]	95.58 [77–115]	126.6 [98-155.8]	193.8 [140-271.7]	χ²=5099.2, *p* < 0.001
HDL-C, mg/dl	49.48 [40–60]	58.76 [50-68.81]	52.58 [44-61.86]	47.17 [40-55.67]	39.82 [32-47.55]	χ²=2150.0, *p* < 0.001
LDL-C, mg/dl	115.6 [94–138]	110.6 [92-128.8]	117.9 [97-138.8]	120.6 [99.55–143]	115.2 [90-143.4]	χ²=127.06, *p* < 0.001
Scr, mg/dl	0.76 [0.66–0.88]	0.75 [0.64–0.86]	0.755 [0.64–0.88]	0.77 [0.66–0.89]	0.77 [0.67–0.9]	χ²=61.08, *p* < 0.001
SUA, mg/dl	4.31 [3.57–5.168]	4 [3.35–4.75]	4.19 [3.52–5.05]	4.38 [3.685–5.25]	4.66 [3.84–5.58]	χ²=370.25, *p* < 0.001
BUN, mg/dl	15.13 [12.6-18.23]	15.52 [12-18.86]	15.24 [12-18.29]	14.99 [13-18.09]	14.82 [12-17.65]	χ²=33.59, *p* < 0.001
CRP, mg/l	1.04 [0.55–2.178]	0.48 [0.34–0.73]	0.83 [0.54–1.4]	1.36 [0.81–2.48]	2.69 [1.38–5.71]	χ²=3728.1, *p* < 0.001
SBP, mmHg	128 [115.3-143.7]	122.7 [111-136.7]	126.7 [114-141.2]	129.7 [117-145.2]	134.3 [120-148.7]	χ²=351.23, *p* < 0.001
DBP, mmHg	75.33 [67.67-84]	72.67 [66-80.67]	74.33 [67-82.33]	76 [68.67–84.67]	78.33 [70.67-87]	χ²=283.86, *p* < 0.001
Diabetes						χ²=928.12, *p* < 0.001
No	3733 (40.1%)	1417 (60.0%)	1091 (46.8%)	812 (35.1%)	413 (17.9%)	
Yes	5569 (59.9%)	943 (40.0%)	1239 (53.2%)	1499 (64.9%)	1888 (82.1%)	
Hypertension						χ²=409.28, *p* < 0.001
No	5135 (55.2%)	1617 (68.5%)	1410 (60.5%)	1170 (50.6%)	938 (40.8%)	
Yes	4167 (44.8%)	743 (31.5%)	920 (39.5%)	1141 (49.4%)	1363 (59.2%)	
HeartDisease						χ²=73.26, *p* < 0.001
No	8054 (86.6%)	2120 (89.8%)	2062 (88.5%)	1988 (86.0%)	1884 (81.9%)	
Yes	1248 (13.4%)	240 (10.2%)	268 (11.5%)	323 (14.0%)	417 (18.1%)	
Dyslipidemia						χ²=2359.52, *p* < 0.001
No	4772 (51.3%)	1919 (81.3%)	1541 (66.1%)	970 (42.0%)	342 (14.9%)	
Yes	4530 (48.7%)	441 (18.7%)	789 (33.9%)	1341 (58.0%)	1959 (85.1%)	
Residence						χ²=115.97, *p* < 0.001
Urban	3456 (37.2%)	722 (30.6%)	789 (33.9%)	918 (39.7%)	1027 (44.6%)	
Rural	5846 (62.8%)	1638 (69.4%)	1541 (66.1%)	1393 (60.3%)	1274 (55.4%)	
Education level						χ²=13.52, *p* = 0.141
Illiterate	2712 (29.2%)	690 (29.2%)	689 (29.6%)	676 (29.3%)	657 (28.6%)	
Primary	3726 (40.1%)	948 (40.2%)	973 (41.8%)	912 (39.5%)	893 (38.8%)	
Second/High school	2732 (29.4%)	696 (29.5%)	641 (27.5%)	685 (29.6%)	710 (30.9%)	
College	132 (1.4%)	26 (1.1%)	27 (1.2%)	38 (1.6%)	41 (1.8%)	
Marital status						χ²=16.91, *p* = 0.050
Never	69 (0.7%)	26 (1.1%)	12 (0.5%)	14 (0.6%)	17 (0.7%)	
Married	8197 (88.1%)	2101 (89.0%)	2060 (88.4%)	2015 (87.2%)	2021 (87.8%)	
Divorced	98 (1.1%)	30 (1.3%)	22 (0.9%)	26 (1.1%)	20 (0.9%)	
Widowed	938 (10.1%)	203 (8.6%)	236 (10.1%)	256 (11.1%)	243 (10.6%)	
Smoking status						χ²=28.54, *p* < 0.001
Never	5735 (61.7%)	1417 (60.0%)	1418 (60.9%)	1441 (62.4%)	1459 (63.4%)	
Current	2748 (29.5%)	764 (32.4%)	718 (30.8%)	658 (28.5%)	608 (26.4%)	
Former	819 (8.8%)	179 (7.6%)	194 (8.3%)	212 (9.2%)	234 (10.2%)	
Drinking status						χ²=18.11, *p* = 0.006
Never	5650 (60.7%)	1362 (57.7%)	1406 (60.3%)	1448 (62.7%)	1434 (62.3%)	
Current	2890 (31.1%)	807 (34.2%)	731 (31.4%)	674 (29.2%)	678 (29.5%)	
Former	762 (8.2%)	191 (8.1%)	193 (8.3%)	189 (8.2%)	189 (8.2%)	
Drinking frequency						χ²=32.73, *p* < 0.001
None	6200 (66.7%)	1487 (63.0%)	1541 (66.1%)	1586 (68.6%)	1586 (68.9%)	
Occasional	1272 (13.7%)	365 (15.5%)	315 (13.5%)	300 (13.0%)	292 (12.7%)	
Weekly	566 (6.1%)	135 (5.7%)	145 (6.2%)	139 (6.0%)	147 (6.4%)	
Near-daily / Daily	1264 (13.6%)	373 (15.8%)	329 (14.1%)	286 (12.4%)	276 (12.0%)	

Test of significance: Kruskal–Wallis test for continuous variables and χ² test for categorical variables

Abbreviations: *CTI* C-reactive protein–triglyceride–glucose index, *Q1–Q4* Quartiles of CTI, *BMI* Body mass index, *WC* Waist circumference, *FPG* Fasting plasma glucose, *HbA1c* Glycated hemoglobin, *TC* Total cholesterol, *TG* Triglycerides, *HDL-C* High-density lipoprotein cholesterol, *LDL-C* Low-density lipoprotein cholesterol, *Scr* Serum creatinine, *SUA* Serum uric acid, *BUN* Blood urea nitrogen, *CRP* C-reactive protein, *SBP* Systolic blood pressure, *DBP* Diastolic blood pressure

### Association between CTI and the risk of LD

Model diagnostics revealed no significant multicollinearity among adjustment covariates (all variance inflation factor [VIF] 1.00–2.07), and lipid variables were pre-screened to prevent redundancy with CTI; comprehensive VIF and correlation matrices are provided in Supplementary Tables S5-6. In the primary Cox analyses, higher CTI levels were independently correlated with increased LD risk (Table 2). When modeled as a continuous variable, each 1-unit increase in CTI correlated with a higher hazard of LD in all models, with the fully adjusted Model 3 yielding an HR of 1.18 (95% CI 1.07–1.30). When modeled in quartiles, participants in Q4 exhibited a significantly higher risk compared with Q1 in Model 3 (HR 1.37; 95% CI: 1.08–1.74), demonstrating a significant trend of increasing risk across CTI quartiles. Schoenfeld residuals indicated no significant violations of the proportional hazards assumption (Supplementary Table S7 and Figure S2).

**Table 2:** Association between CTI and incident self-reported physician-diagnosed liver disease: hazard ratios from Cox regression models

Characteristic	Model	1		Model	2		Model	3	
	HR	95%CI	*P*	HR	95%CI	*P*	HR	95%CI	*P*
CTI(per 1unit)	1.22	1.12,1.32	< 0.001	1.22	1.12,1.32	< 0.001	1.18	1.07,1.30	< 0.001
CTI quartile									
Q1	Ref			Ref			Ref		
Q2	1.20	0.97,1.49	0.092	1.22	0.98,1.51	0.075	1.21	0.97,1.50	0.091
Q3	1.23	0.99,1.53	0.058	1.25	1.00,1.55	0.046	1.20	0.95,1.50	0.120
Q4	1.48	1.20,1.82	< 0.001	1.49	1.21,1.84	< 0.001	1.37	1.08,1.74	0.009
P for trend			< 0.001			< 0.001			0.016

Model 1 unadjusted

Model 2 was additionally adjusted for sex, age, residence, educational level, and smoking status

Model 3 was further adjusted for hypertension, heart disease, body mass index (BMI), low-density lipoprotein cholesterol (LDL-C), high-density lipoprotein cholesterol (HDL-C), and drinking frequency

Abbreviations: *HR* Hazard ratio, *CI* Confidence interval, *CTI* CRP–triglyceride–glucose index, *Ref* Reference group

**Fig. 2:**
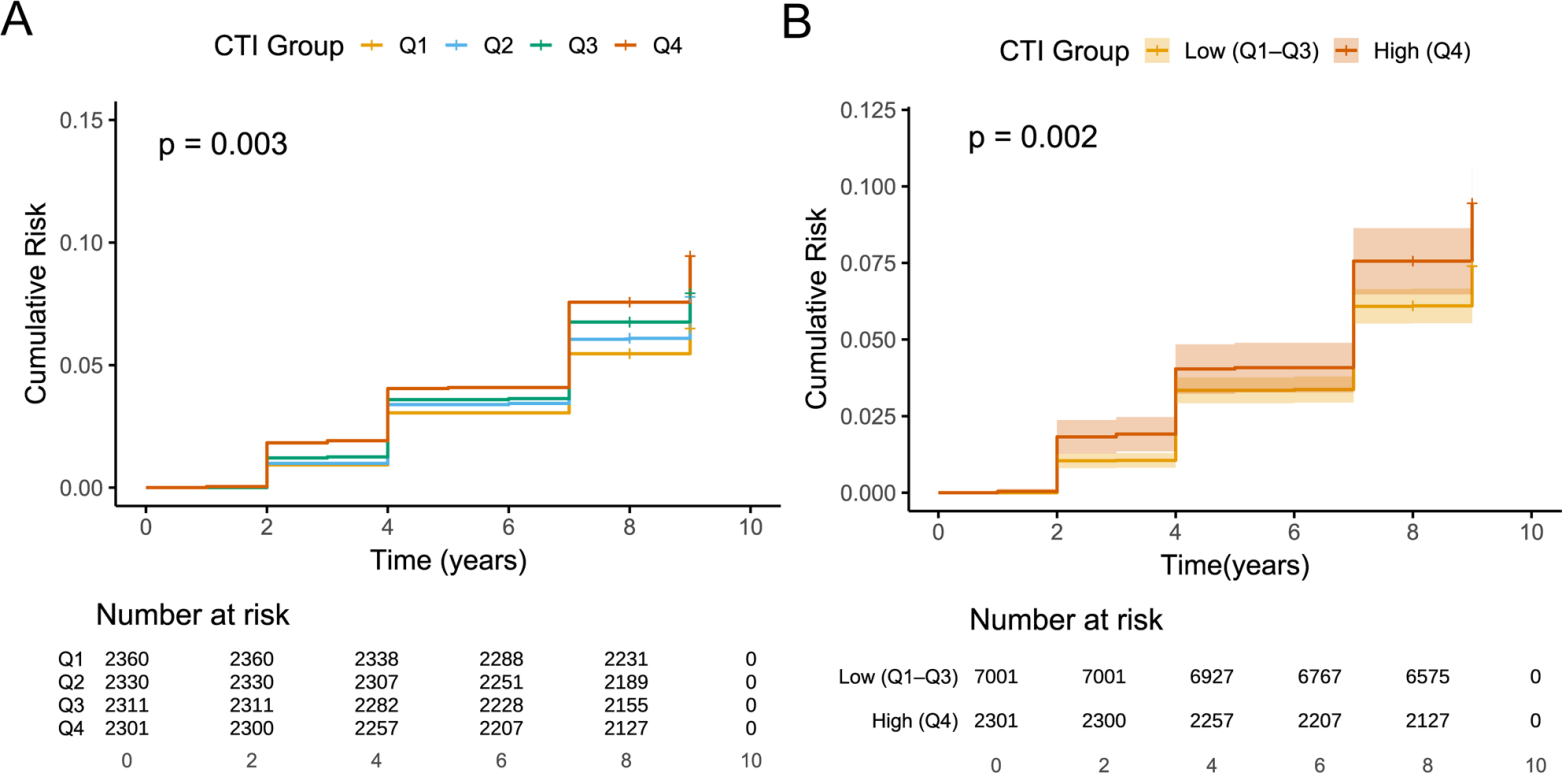
Cumulative incidence of liver-related events by CTI level. **A** Kaplan–Meier survival curves across CTI quartiles. **B** Comparison of cumulative risk between highest quartile (Q4) and pooled lower quartiles (Q1–Q3)

**Fig. 3:**
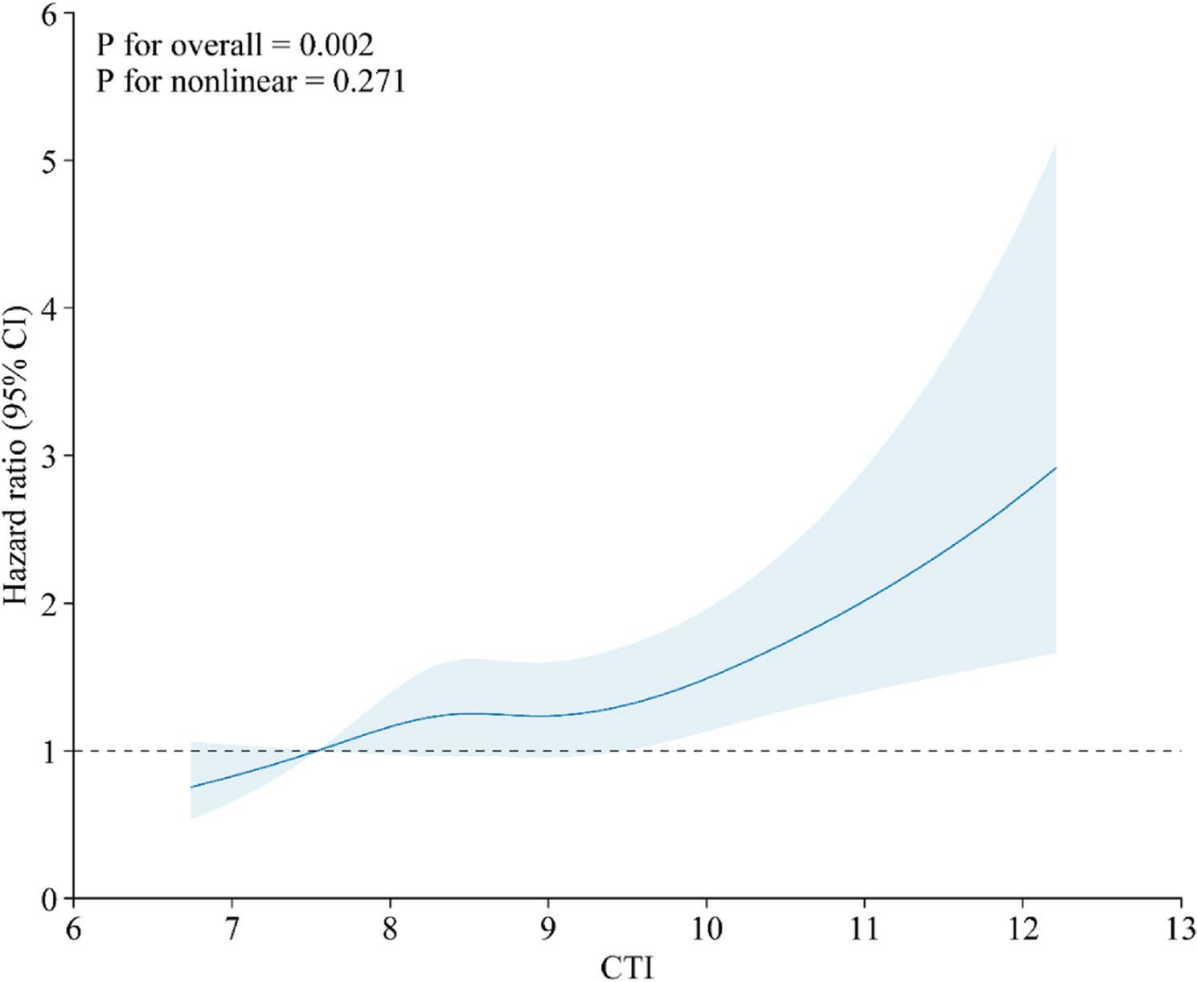
Association between CTI and liver-related outcomes modeled using restricted cubic splines. Models were fully adjusted for demographic, lifestyle, and clinical covariates consistent with the main analyses

Kaplan–Meier curves illustrated a graded increase in cumulative incidence of LD with higher CTI categories (Fig. 2). Individuals in Q4 exhibited consistently higher cumulative incidence than those in Q1–Q3, both when comparing all four quartiles and when contrasting Q4 with Q1–Q3. Restricted cubic spline analyses, treating CTI as a continuous variable, revealed a significant overall association with LD risk, indicating an approximately monotonic dose–response relationship, with minimal evidence of nonlinearity (Fig. 3). To improve clinical interpretability, we employed the fully adjusted Cox model to calculate standardized absolute risks at 5 and 7 years. The 5-year standardized risk of incident self-reported physician-diagnosed LD increased from approximately 3.0% in Q1 to 4.0% in Q4, and the 7-year risk increased from 5.4% to 7.4%, with intermediate estimates for Q2 and Q3 (Supplementary Table S8). These absolute risk gradients aligned with the stepwise increase in HRs across CTI quartiles.

### Subgroup and sex-specific dose–response associations

**Fig. 4:**
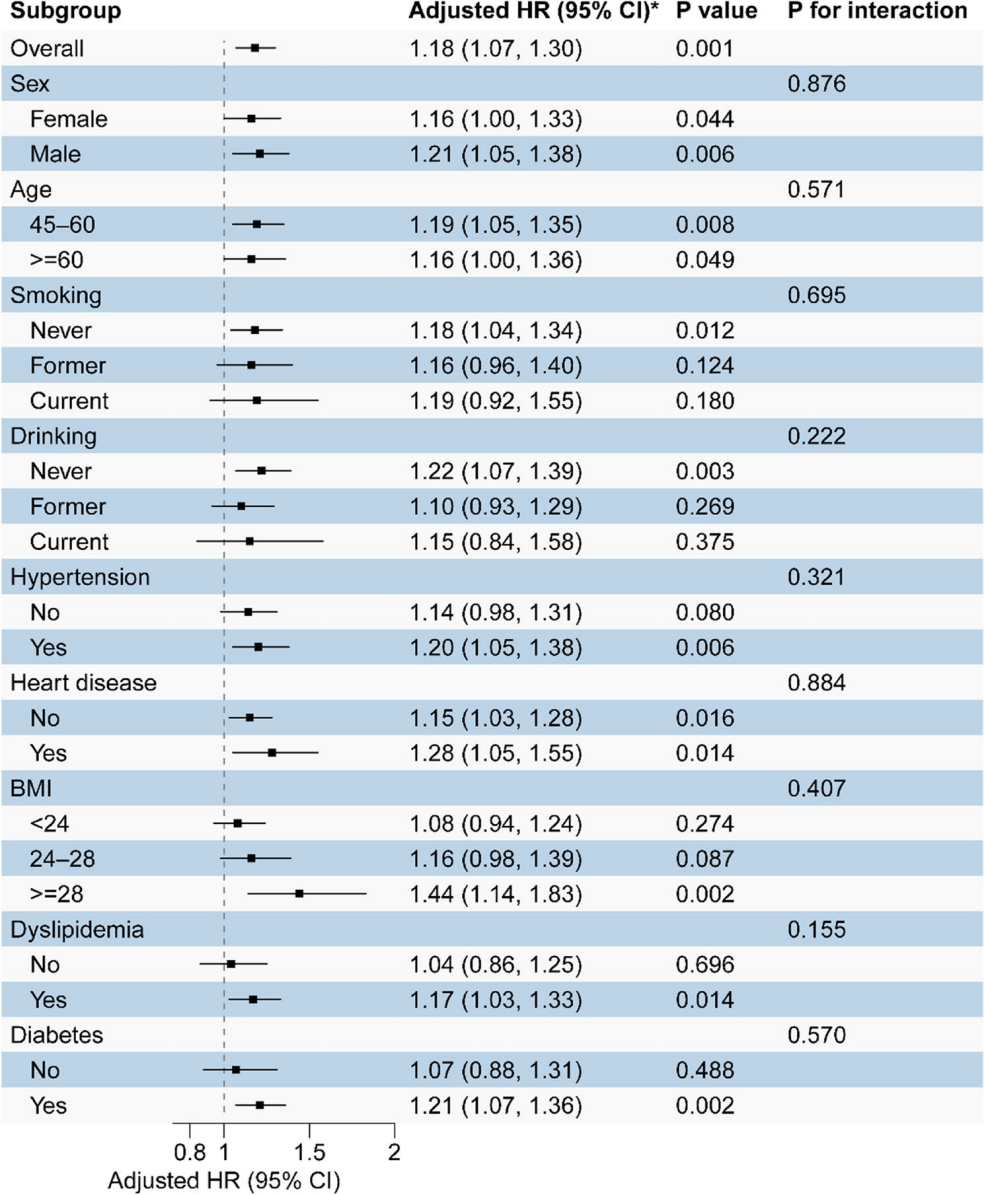
Subgroup-specific associations between CTI and liver-related outcomes. Forest plot shows adjusted hazard ratios (HRs) and 95% confidence intervals per 1-unit increase in CTI across predefined strata. Interaction P values test for effect modification. Models were fully adjusted for demographic, lifestyle, and clinical covariates consistent with the main analyses

Forest plots illustrated prespecified subgroup-specific associations between CTI and incident liver disease across strata of sex, age, smoking and drinking status, hypertension, heart disease, BMI, dyslipidemia, and diabetes (Fig. 4). In all subgroups, higher CTI (per 1-unit increase) was consistently associated with increased LD risk, with no statistically significant multiplicative interactions detected (all *P* for interaction > 0.05), indicating that the negative association between CTI and LD was uniformly comparable across demographic and clinical strata. Given multiplicity, these subgroup/interaction results are interpreted as exploratory and the interaction P values are nominal.

**Fig. 5:**
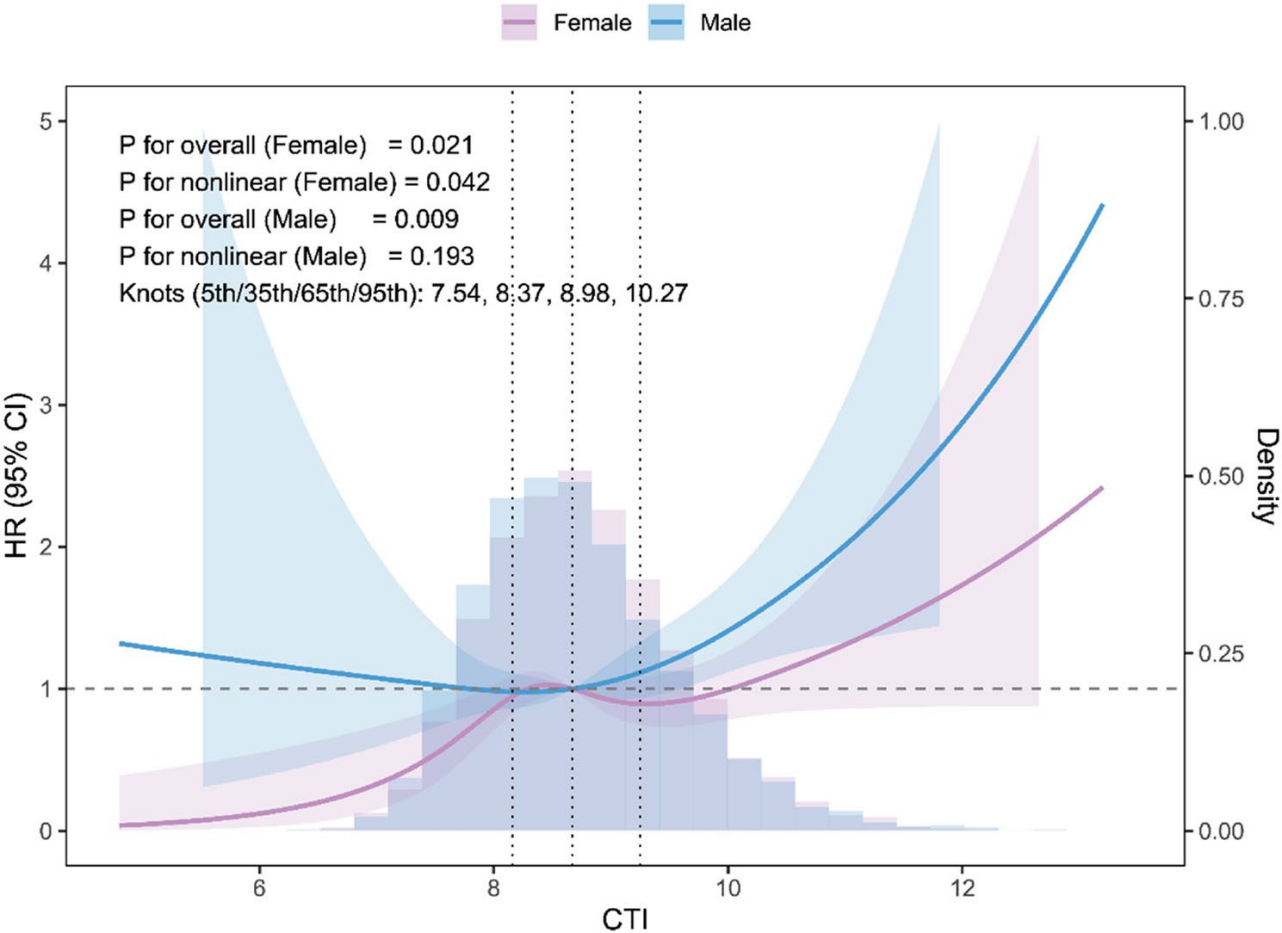
Sex-stratified dose–response analysis of CTI and liver-related event risk. Restricted cubic spline curves illustrate the adjusted hazard ratios and 95% confidence intervals for males and females. Background density plots show the sex-specific CTI distributions. Models were fully adjusted for demographic, lifestyle, and clinical covariates consistent with the main analyses

Sex-stratified Cox and restricted cubic spline models (Table 3; Fig. 5) were employed to investigate potential sex-specific differences in the CTI–LD association. In fully adjusted Cox models, elevated CTI correlated with increased LD risk in both sexes, with broadly comparable effect sizes per 1-unit increase; in quartile analyses, Q4 tended to have higher risk than Q1 in both sexes, although the Q4–Q1 disparity in women was marginal. In the primary four-knot splines (knots at the 5th, 35th, 65th, and 95th percentiles of CTI; 7.54, 8.37, 8.98, and 10.27), elevated CTI was positively correlated with LD risk in both sexes. In sensitivity analyses using three-knot splines with common knots at the 10th, 50th, and 90th percentiles, the overall association between CTI and LD remained statistically significant in men and was borderline in women, and formal tests for nonlinearity were not significant in either sex. The sex-specific curves were approximately monotonic in both sexes, with only slightly steeper slopes at higher CTI values in females, with no robust evidence of distinct nonlinear patterns (Figure S3). Consistent with these findings, the CTI × sex interaction was not statistically significant, indicating comparable effect sizes across sexes, and Schoenfeld residuals did not indicate significant violations of the proportional hazards assumption (Supplementary Table S7).

**Table 3:** Sex-specific associations between CTI and incident self-reported physician-diagnosed liver disease

Sex	Characteristic	Model	1		Model	2		Model	3	
		HR	95%CI	*P*	HR	95%CI	*P*	HR	95%CI	*P*
Male	CTI(per 1unit)	1.21	1.08,1.36	0.001	1.21	1.07,1.35	0.002	1.21	1.05,1.38	0.006
	Q1	Ref			Ref			Ref		
	Q2	1.08	0.80,1.45	0.604	1.08	0.81,1.46	0.597	1.11	0.82,1.50	0.512
	Q3	1.05	0.78,1.43	0.731	1.05	0.78,1.43	0.740	1.04	0.76,1.44	0.791
	Q4	1.51	1.14,2.00	0.004	1.50	1.13,2.00	0.005	1.50	1.09,2.06	0.014
P for trend				0.007			0.008			0.026
Female	CTI(per 1unit)	1.23	1.09,1.38	< 0.001	1.24	1.10,1.40	< 0.001	1.16	1.00,1.33	0.044
	Q1	Ref			Ref			Ref		
	Q2	1.37	1.00,1.87	0.053	1.40	1.02,1.92	0.039	1.33	0.96,1.83	0.084
	Q3	1.46	1.07,1.98	0.017	1.49	1.09,2.04	0.011	1.36	0.98,1.90	0.066
	Q4	1.50	1.10,2.03	0.010	1.54	1.13,2.10	0.006	1.26	0.88,1.79	0.208
P for trend				0.012			0.008			0.285

Sex-stratified Cox proportional hazards regression models were used to estimate hazard ratios (HRs) and 95% confidence intervals (CIs) for liver-related outcomes per 1-unit increase in CTI and by CTI quartiles

Model 1 unadjusted

Model 2 was additionally adjusted for age, residence, educational level, and smoking status

Model 3 was further adjusted for hypertension, heart disease, body mass index (BMI), low-density lipoprotein cholesterol (LDL-C), high-density lipoprotein cholesterol (HDL-C), and drinking frequency

### CTI for incident LD risk: time-dependent AUC and cross-sectional ROC

**Fig. 6:**
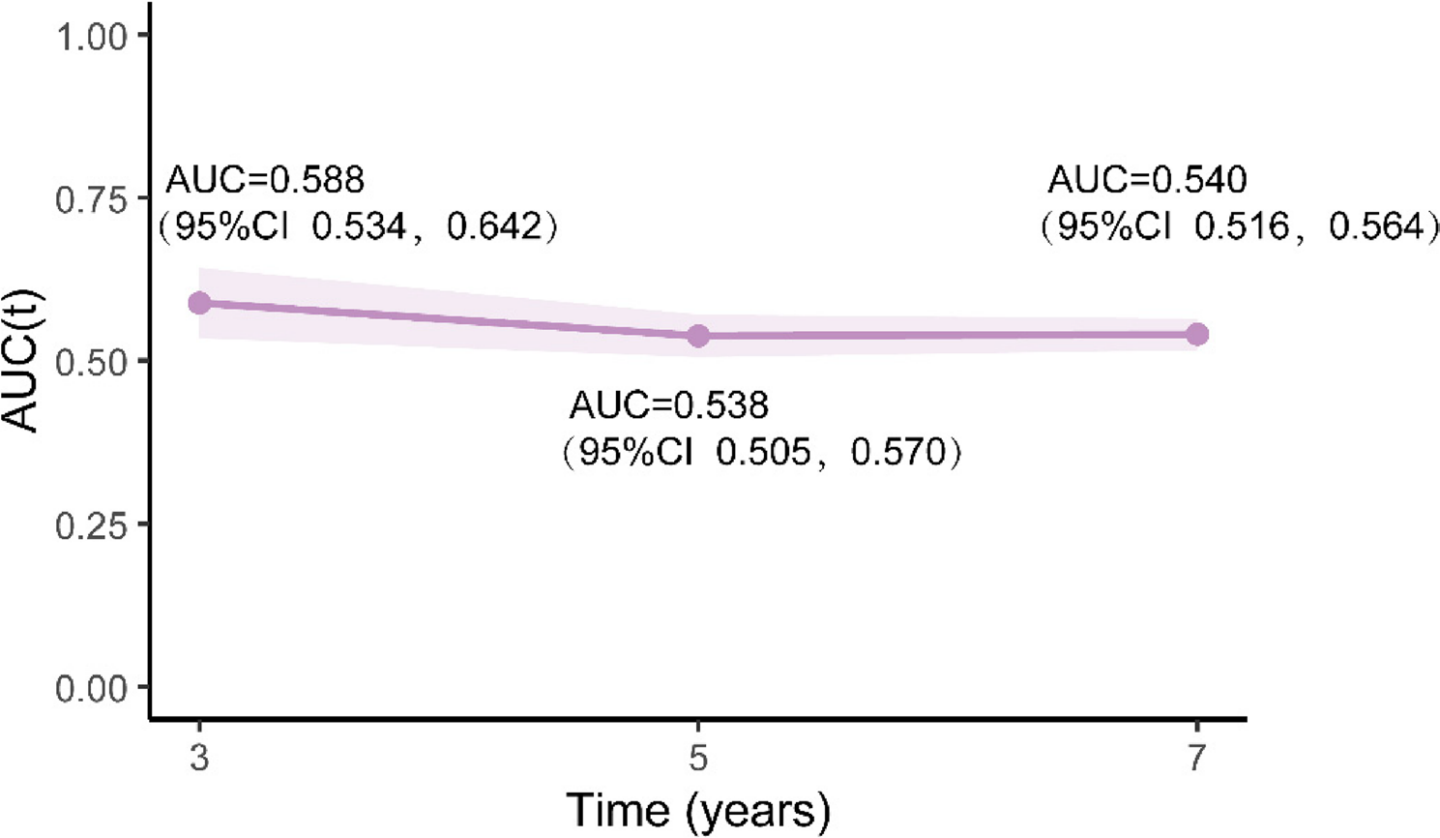
Time-dependent AUC of a CTI for incident liver disease

Time-dependent ROC analyses demonstrated modest discrimination for future LD by CTI alone, with AUC(t) values consistent in the mid-0.5 range at 3, 5, and 7 years (Fig. 6). In cross-sectional ROC analyses, CTI demonstrated similar discriminative capability to TG and slightly better performance than CRP (Figure S4 and Supplementary Table S9). Adding CTI to CRP + TG did not significantly change AUC but enhanced overall model fit in nested Cox models, indicating that CTI encompasses complementary risk information beyond its individual components.

### Role of non-TG dyslipidemia in the CTI–LD association

**Fig. 7:**
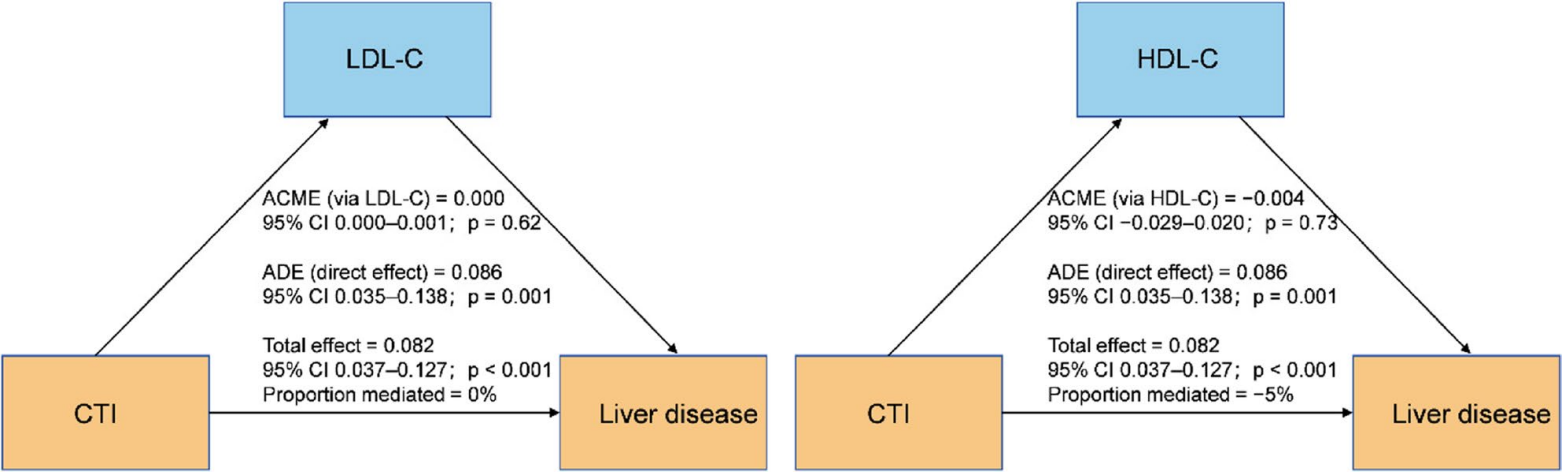
Mediation analysis of the CTI–liver disease association: LDL-C (A) and HDL-C (B). Reported are the average causal mediation effect (ACME), average direct effect (ADE), and proportion mediated. Models were fully adjusted for demographic, lifestyle, and clinical covariates consistent with the main analyses

In the fully adjusted total-effect model that excluded LDL-C and HDL-C (Model 3_total), elevated CTI was correlated with an increased risk of incident LD, exhibiting a clear gradient across CTI quartiles. Incorporating LDL-C and HDL-C into the direct-effect model (Model 3_direct) resulted in only marginal alterations to the estimated hazard ratios for CTI, suggesting that additional adjustment for these lipoproteins has a negligible effect on the CTI–LD association (Table S10). Consistent with this, causal mediation analyses revealed that LDL-C and HDL-C accounted for virtually none of the relationship between CTI and LD: the mediated effects through either lipoprotein were close to zero, whereas the direct effect of CTI remained robust and statistically significant (Fig. 7). Overall, these findings indicate that the excess LD risk associated with higher CTI is driven largely by pathways other than LDL-C and HDL-C.

### Sensitivity analyses

Sensitivity analyses consistently affirmed the robustness of the primary findings. Excluding participants with elevated baseline CRP, early incident LD, or high-frequency drinkers yielded CTI effect estimates that closely mirrored the magnitude and direction of those of the main analysis, maintaining positive trends across CTI quartiles (Supplementary Tables S11–13). Re-estimating the models using attained age as the underlying time scale and applying Fine–Gray competing-risk models with death without LD as a competing event yielded results that closely mirrored the primary Cox models (Supplementary Tables S14-15). Re-defining event times using a mid-interval approximation (assigning incident LD to the midpoint between two survey waves) exhibited a negligible impact on the estimated CTI–LD association (Supplementary Table S16).

To assess potential bias from loss to follow-up, we compared the baseline characteristics of participants included in the analytic cohort with those excluded due to missing follow-up; individuals without follow-up exhibited modest differences but no significant imbalances across key characteristics (Table S17). In an inverse-probability-of-censoring weighting analysis that up-weighted participants with baseline profiles similar to those without follow-up, the CTI–LD association remained nearly identical to the unweighted estimate (Table S18), indicating that differential loss to follow-up is improbable to have significantly distorted the results. Employing a more rigorous endpoint that necessitated simultaneous self-reporting of physician diagnosis and liver-disease medication use resulted in slightly stronger but broadly consistent associations with CTI (Table S19), whereas baseline profiles were generally similar between medicated and non-medicated cases (Table S20). Analyses restricted to participants with repeated CTI measurements revealed patterns that were markedly concordant with those identified in the overall cohort (Table S21). Finally, as a quantitative bias analysis to bound potential unmeasured confounding, we calculated E-values for the primary associations. For the fully adjusted association per 1-unit increase in CTI (HR 1.18; 95% CI 1.07–1.30), the E-value was 1.64 (E-value for the lower CI limit, 1.34). For the Q4 versus Q1 contrast (HR 1.37; 95% CI 1.08–1.74), the E-value was 2.08 (lower-limit E-value, 1.37), indicating that an unmeasured confounder would need to be associated with both CTI and incident liver disease by risk ratios of at least these magnitudes, conditional on measured covariates, to fully explain away the observed associations.

## Discussion

In this extensive, prospective, nationally representative cohort of middle-aged and older Chinese adults, higher values of the CTI index were associated with an increased risk of incident self-reported physician-diagnosed LD, irrespective of demographic, behavioral, and cardiometabolic factors. The association was approximately linear in both sexes and generally comparable in magnitude, exhibiting only modest evidence of sex-specific differences in dose–response shape.

These findings are biologically credible. Insulin resistance and low-grade systemic inflammation are fundamental mechanisms in the onset and progression of LD. CTI concurrently measures these processes by combining a surrogate of insulin resistance (TG–glucose) with a systemic inflammatory marker (CRP). Experimental and clinical data indicate that insulin resistance facilitates hepatic lipid accumulation, lipotoxicity, and mitochondrial dysfunction, which in turn triggers oxidative and endoplasmic reticulum stress and activates pro-inflammatory pathways such as c-Jun N-terminal kinase [18, 19]. Simultaneously, chronic liver damage is sustained by innate immune activation, danger- and pathogen-associated molecular patterns, and inflammasome signaling, which promote stellate cell activation, extracellular matrix deposition, and fibrogenesis [20—22]. As a systemic inflammatory biomarker, CRP is associated with fibrosis progression and adverse hepatic outcomes [23, 24], whereas TyG and related indices monitor metabolic dysfunction and correlate with mortality in MASLD [11, 12]. By integrating both axes, CTI may provide a more comprehensive overview of metabolic-inflammatory stress than either component independently.

Our results build upon previous research that has assessed insulin resistance and inflammatory markers independently [25, 26]. Using CTI rather than TyG or CRP in isolation highlights their complementary insights: triglyceride–glucose indicates insulin-resistance–related metabolic stress, and CRP signifies systemic inflammation [27]. Collectively, they may effectively encapsulate the multidimensional burden of cardiometabolic dysfunction relevant to hepatic steatosis, necro-inflammation, and fibrosis. To our knowledge, this is the first study to evaluate CTI in relation to LD risk in a general population, thereby expanding its clinical applicability beyond cardiovascular and cancer outcomes and supporting CTI as an integrated marker of metabolic and inflammatory liver susceptibility.

Sex-stratified analyses revealed that elevated CTI correlated with increased LD risk in men and women, exhibiting effect sizes that were broadly comparable and lacking statistically significant interaction. The spline-based dose–response curves indicated an approximately linear increase in risk, with only modest deviation from linearity in women. These patterns are consistent with accumulating evidence that biological sex influences LD susceptibility [28—32], but our findings indicate more quantitative than qualitative differences in CTI-related risk. From a practical standpoint, “sex should be considered when interpreting CTI-based risk stratification”; however, current data do not support fundamentally different risk thresholds based on sex. Future research with etiologically characterized cohorts, more events, and comprehensive hormonal and body-fat distribution measures is necessary to clarify if sex-specific cut-offs or prediction models significantly enhance risk classification.

Mediation analyses offered additional understanding of the underlying mechanisms. In both the probit-based parallel-mediator structural equation model and the counterfactual single-mediator analyses, LDL-C or HDL-C did not meaningfully mediate the CTI–LD association: the indirect effects through these lipoproteins were essentially null, whereas the direct effect of CTI remained statistically significant. One interpretation is that the triglyceride component of CTI—which is closely associated with insulin resistance, *de novo* lipogenesis, and ectopic fat deposition—and the CRP component—indicative of low-grade systemic inflammation—encapsulates hepatotoxic processes inadequately represented by conventional LDL-C and HDL-C levels. Simultaneously, these null mediation results should be interpreted cautiously because LDL-C and HDL-C were measured only once, any true mediating effects may be minimal, and unmeasured lipid fractions or measurement error could attenuate estimates. Overall, the data indicate that non-triglyceride lipoprotein pathways (LDL-C/HDL-C) do not significantly explain the observed association and that insulin-resistance–related metabolic stress and systemic inflammation, as collectively measured by CTI, are likely to be the dominant mechanisms.

From a public-health perspective, CTI has several attractive features. It is derived from three commonly measured, low-cost biomarkers (CRP, TG, and fasting glucose); does not necessitate imaging or specialized apparatus; and can be opportunistically calculated in primary care or community settings where full liver panels and platelet counts are not consistently available. Despite modest discrimination for individual-level prediction, CTI consistently identified groups with elevated absolute risk and indicated a graded increase in incidence rates and standardized absolute risks across quartiles. In this context, CTI may potentially serve as a population-level, cost-effective risk indicator rather than a stand-alone screening test to identify individuals who warrant closer liver evaluation, lifestyle counseling, or targeted metabolic risk management, particularly in aging populations with high burdens of metabolic risk factors.

This study has some limitations. First, the outcome was incident self-reported physician-diagnosed LD, defined from participants’ reports of previous diagnosis or treatment. This extensive, self-reported endpoint is susceptible to misclassification, recall, and detection bias, and it does not allow etiologic subtyping (MASLD versus viral, alcohol-related, autoimmune, or drug-induced LD). Non-differential under-ascertainment of steatotic LD, particularly MASLD, would tend to bias the CTI association toward the null. Second, numerous significant potential confounders could not be fully captured. CHARLS excludes serologic markers of hepatitis B or C (HBsAg/anti-HCV), physician-diagnosed hepatitis with etiologic classification, or transfusion history, thus precluding adjustments for viral hepatitis or its inclusion in a formal quantitative bias analysis. For alcohol, we adjusted for a recoded four-level measure of current drinking frequency as a pragmatic proxy for dosage, but more granular consumption data were not available. Physical activity was assessed only through MET-min per week and exhibited a high proportion of missing values (~ 63%), precluding its inclusion as a covariate without strong, unverifiable assumptions. Therefore, residual confounding from viral hepatitis, specific drinking patterns, and physical activity cannot be ruled out.

Moreover, CTI was measured solely at baseline, preventing examination of long-term exposure or temporal variations, and the cohort primarily comprised middle-aged and older Chinese adults, potentially restricting generalizability to younger populations and other ethnic groups. Therefore, the observed association should be interpreted as reflecting baseline CTI and may underestimate the impact of long-term exposure to elevated metabolic-inflammatory burden. Future studies should evaluate CTI trajectories and consider time-varying confounding due to changes in cardiometabolic risk factors and treatments over follow-up. Aspartate aminotransferase, alanine aminotransferase, and platelet counts were unavailable, precluding the calculations of FIB-4 or APRI or directly benchmarking CTI against guideline-recommended non-invasive fibrosis scores. External validation in datasets with comprehensive liver phenotyping and laboratory panels will therefore be crucial to define CTI’s additional clinical value.

## Conclusion

CTI, a composite marker of systemic inflammation and insulin resistance, was independently correlated with incident self-reported physician-diagnosed LD in middle-aged and older Chinese adults, exhibiting broadly similar, approximately linear associations in men and women. As a simple, economical index derived from routine laboratory tests, CTI may complement existing non-invasive tools by facilitating opportunistic, population-level risk stratification, especially where full liver panels are unavailable.

## Supplementary Information

Supplementary Material 1.

## Data Availability

The data supporting the findings of this study are available on the CHARLS website (http://charls.pku.edu.cn/).
