# Supplementary material for: Association of the C-reactive protein–triglyceride–glucose index with liver disease risk: findings from a nationwide Chinese cohort
Source: BMC Gastroenterol. 2026 Jan 3;26:87. doi: 10.1186/s12876-025-04588-2 (PMC12866445; doi:10.1186/s12876-025-04588-2)
Supplement: Supplementary file 1 — Supplementary Material 1. [file 12876_2025_4588_MOESM1_ESM.docx]

**Supplementary Methods**

**S1. Proportional hazards and collinearity diagnostics**

The proportional hazards assumption was assessed using Schoenfeld residuals, including both global and covariate-specific tests. Multicollinearity was evaluated using variance inflation factors (VIFs). Correlation matrices among lipid-related variables and CTI components were examined to avoid redundancy.

**S2. Multiple imputation**

Missing covariate data were handled using multiple imputation by chained equations (MICE) with m = 30 imputations and 20 iterations. Variable-appropriate imputation models were used (e.g., predictive mean matching for continuous variables and logistic/multinomial models for categorical variables). The imputation model included the outcome indicator, follow-up time, CTI (continuous), and all covariates used in the analytic models. Imputed datasets were analyzed separately and pooled using Rubin’s rules. Complete-case analyses were conducted as a robustness check.

**S3. Sensitivity analyses**

We performed prespecified sensitivity analyses to test the robustness of the primary findings, including: (i) using attained age as the underlying time scale; (ii) Fine–Gray subdistribution hazard models with death without liver disease as a competing event; (iii) inverse-probability-of-censoring weighting (IPCW), with stabilized weights derived from a logistic regression model predicting having follow-up vs being lost to follow-up based on baseline covariates; (iv) mid-interval event time assignment (assigning incident liver disease to the midpoint between two survey waves); (v) excluding participants with marked inflammation at baseline (e.g., CRP > 10 mg/L) and excluding early events within prespecified periods; (vi) excluding high-frequency drinkers / high-risk alcohol consumption; (vii) restricting analyses to participants with repeated measurements of CTI components in later waves; and (viii) using a more stringent endpoint requiring both physician diagnosis and liver-disease medication use, and descriptively comparing medicated versus non-medicated cases.

**S4. Quantitative bias analysis: E-values**

**E-values** were calculated to quantify the minimum strength of association, on the **risk-ratio scale**, that an unmeasured confounder would need to have with both the exposure (CTI) and the outcome (incident self-reported physician-diagnosed liver disease), above and beyond the measured covariates, to fully explain away the observed associations. E-values were computed for estimates from the **fully adjusted primary model (Model 3)**, treating hazard ratios (HRs) as approximations to risk ratios.

For an estimated association **RR (or HR) ≥ 1**, the E-value is:

$$E\text{-value}=RR+\sqrt{RR\times(RR-1)}.$$

For the lower confidence limit **L ≥ 1**, the E-value for the confidence interval is:

$$E\text{-value}_{CI}=L+\sqrt{L\times(L-1)}.$$

**S5. Software**

All statistical analyses were conducted in R version 4.4.0. To support reproducibility, we provide the full analysis code as module-based R scripts in the Supplementary Code Appendix. The exact software environment is documented by exporting package names and versions (including, but not limited to, riskRegression, pROC, lavaan, and related dependencies) using sessionInfo() and packageVersion().

**Table S1.** Overview of variables and missingness in the multiple imputation model

| Variable | MICE method | Role | Type | No. of missing values (n) | Percentage missing (%) |
| --- | --- | --- | --- | --- | --- |
| Sleeping time | pmm | imputed/predictor | continuous | 422 | 4.54 |
| Height | pmm | imputed/predictor | continuous | 1374 | 14.77 |
| Weight | pmm | imputed/predictor | continuous | 1349 | 14.5 |
| BMI | pmm | imputed/predictor | continuous | 1393 | 14.98 |
| WC | pmm | imputed/predictor | continuous | 1346 | 14.47 |
| SBP | pmm | imputed/predictor | continuous | 1358 | 14.6 |
| DBP | pmm | imputed/predictor | continuous | 1357 | 14.59 |
| HbA1c | pmm | imputed/predictor | continuous | 74 | 0.8 |
| LDL_C | pmm | imputed/predictor | continuous | 16 | 0.17 |
| Scr | pmm | imputed/predictor | continuous | 4 | 0.04 |
| BUN | pmm | imputed/predictor | continuous | 1 | 0.01 |
| Hypertension | logreg | imputed/predictor | binary | 946 | 10.17 |
| Smoking | polyreg | imputed/predictor | multicategory | 176 | 1.89 |
| Alcohol | polyreg | imputed/predictor | multicategory | 13 | 0.14 |
| HeartDisease | logreg | imputed/predictor | binary | 23 | 0.25 |
| Education | polyreg | imputed/predictor | multicategory | 3 | 0.03 |
| Drinking frequency | polyreg | imputed/predictor | multicategory | 552 | 5.93 |
| FPG | pmm | imputed/predictor | continuous | 0 | 0 |
| TC | pmm | imputed/predictor | continuous | 0 | 0 |
| TG | pmm | imputed/predictor | continuous | 0 | 0 |
| HDL_C | pmm | imputed/predictor | continuous | 0 | 0 |
| SUA | pmm | imputed/predictor | continuous | 0 | 0 |
| CRP | pmm | imputed/predictor | continuous | 0 | 0 |
| Diabetes | logreg | imputed/predictor | binary | 0 | 0 |
| Age | pmm | imputed/predictor | continuous | 0 | 0 |
| Residence | logreg | imputed/predictor | binary | 0 | 0 |
| Sex | logreg | imputed/predictor | binary | 0 | 0 |
| Marital | polyreg | imputed/predictor | multicategory | 0 | 0 |
| Dyslipidemia | logreg | imputed/predictor | binary | 0 | 0 |
| CTI |  | predictor_only (not imputed) | NA | NA | NA |

For each variable included in the multiple imputation model, the table reports the variable type, imputation method used in MICE, its role in the imputation model, and the number and percentage of missing values before imputation. “Type” indicates whether the variable was treated as continuous, binary, or multicategory. “MICE method” refers to the univariate imputation model used for that variable (pmm = predictive mean matching; logreg = logistic regression; polyreg = polytomous logistic regression). “Role” specifies whether the variable was both imputed and used as a predictor (“imputed/predictor”) or used only as a predictor (“predictor_only”). Missing percentages are calculated relative to the total analytic sample size before imputation.

**Table S2** Comparison of Cox regression analyses between multiple imputation and complete-case datasets

| Source | Covariate | HR | CI_low | CI_high | p.value |
| --- | --- | --- | --- | --- | --- |
| MI (m=30) | CTI | 1.179 | 1.070 | 1.299 | 0.001 |
|  | Sex: male vs female | 1.201 | 0.960 | 1.502 | 0.108 |
|  | Age | 0.987 | 0.978 | 0.996 | 0.005 |
|  | BMI | 0.999 | 0.995 | 1.004 | 0.742 |
|  | Smoking status |  |  |  |  |
|  | current vs never | 0.756 | 0.605 | 0.944 | 0.014 |
|  | former vs never | 1.188 | 0.908 | 1.554 | 0.208 |
|  | Hypertension: yes vs no | 1.132 | 0.967 | 1.326 | 0.122 |
|  | HeartDisease: yes vs no | 2.051 | 1.717 | 2.449 | 0.000 |
|  | LDL_C | 0.999 | 0.997 | 1.001 | 0.514 |
|  | Residence: rural vs urban | 1.077 | 0.920 | 1.260 | 0.355 |
|  | Education |  |  |  |  |
|  | primary vs illiterate | 1.184 | 0.973 | 1.439 | 0.091 |
|  | second/high school vs illiterate | 1.145 | 0.913 | 1.436 | 0.241 |
|  | college vs illiterate | 1.027 | 0.547 | 1.930 | 0.933 |
|  | HDL_C | 1.001 | 0.995 | 1.007 | 0.689 |
|  | Drinking frequency |  |  |  |  |
|  | occasional vs none | 1.122 | 0.695 | 1.810 | 0.638 |
|  | weekly vs none | 1.085 | 0.573 | 2.054 | 0.803 |
|  | near-daily / daily vs none | 1.287 | 0.723 | 2.289 | 0.391 |
| Complete-case (n=7414) | CTI | 1.178 | 1.054 | 1.318 | 0.004 |
|  | Sex: male vs female | 1.116 | 0.865 | 1.440 | 0.399 |
|  | Age | 0.990 | 0.980 | 1.000 | 0.062 |
|  | BMI | 0.999 | 0.995 | 1.004 | 0.751 |
|  | Smoking status |  |  |  |  |
|  | current vs never | 0.750 | 0.582 | 0.967 | 0.027 |
|  | former vs never | 1.267 | 0.942 | 1.705 | 0.118 |
|  | Hypertension: yes vs no | 1.112 | 0.936 | 1.322 | 0.227 |
|  | HeartDisease: yes vs no | 1.943 | 1.591 | 2.372 | 0.000 |
|  | LDL_C | 0.999 | 0.997 | 1.002 | 0.550 |
|  | Residence: rural vs urban | 1.095 | 0.917 | 1.308 | 0.316 |
|  | Education |  |  |  |  |
|  | primary vs illiterate | 1.215 | 0.977 | 1.511 | 0.079 |
|  | second/high school vs illiterate | 1.198 | 0.928 | 1.546 | 0.165 |
|  | college vs illiterate | 1.199 | 0.551 | 2.609 | 0.648 |
|  | HDL_C | 1.000 | 0.994 | 1.007 | 0.954 |
|  | Drinking frequency |  |  |  |  |
|  | occasional vs none | 1.283 | 0.772 | 2.131 | 0.336 |
|  | weekly vs none | 1.388 | 0.709 | 2.718 | 0.339 |
|  | near-daily / daily vs none | 1.302 | 0.703 | 2.412 | 0.401 |
| MI (m=30) | Events | 734 |  |  |  |
| Complete-case (n=7414) | Events | 579 |  |  |  |
| MI (m=30) | Person_time | 81229 |  |  |  |
| Complete-case (n=7414) | Person_time | 64772 |  |  |  |

For the MI analysis, missing covariate values were imputed using multiple imputation by chained equations, and model estimates were pooled across imputations using Rubin’s rules. The same covariate adjustment set was applied in both analyses. HRs for continuous variables are expressed per 1-unit increase unless otherwise specified; categorical variables are compared with the indicated reference category. MI, multiple imputation; HR, hazard ratio; CI, confidence interval.

**Table S3.** Fully adjusted multivariable Cox regression after multiple imputation

| Variable | HR | CI_low | CI_high | p.value | FMI | Monte-Carlo error |
| --- | --- | --- | --- | --- | --- | --- |
| CTI | 1.180 | 1.071 | 1.301 | 0.001 | 0.005 | <0.001 |
| Sex: male vs female | 1.201 | 0.960 | 1.502 | 0.109 | 0.011 | 0.002 |
| Age | 0.987 | 0.978 | 0.996 | 0.005 | 0.006 | <0.001 |
| BMI | 0.999 | 0.995 | 1.004 | 0.759 | 0.144 | <0.001 |
| Smoking status |  |  |  |  |  |  |
| current vs never | 0.759 | 0.607 | 0.948 | 0.015 | 0.022 | 0.003 |
| former vs never | 1.201 | 0.920 | 1.569 | 0.178 | 0.029 | 0.004 |
| Hypertension: yes vs no | 1.127 | 0.962 | 1.320 | 0.140 | 0.067 | 0.004 |
| HeartDisease: yes vs no | 2.051 | 1.718 | 2.449 | 0.000 | 0.011 | 0.001 |
| LDL_C | 0.999 | 0.997 | 1.001 | 0.510 | 0.003 | <0.001 |
| Residence: rural vs urban | 1.076 | 0.920 | 1.260 | 0.358 | 0.003 | <0.001 |
| Education |  |  |  |  |  |  |
| primary vs illiterate | 1.183 | 0.972 | 1.438 | 0.093 | 0.003 | <0.001 |
| second/high school vs illiterate | 1.145 | 0.913 | 1.436 | 0.241 | 0.003 | <0.001 |
| college vs illiterate | 1.024 | 0.545 | 1.925 | 0.940 | 0.004 | 0.002 |
| HDL_C | 1.001 | 0.996 | 1.007 | 0.656 | 0.006 | <0.001 |
| Drinking frequency |  |  |  |  |  |  |
| occasional vs none | 1.119 | 0.693 | 1.806 | 0.646 | 0.007 | 0.003 |
| weekly vs none | 1.020 | 0.544 | 1.914 | 0.950 | 0.085 | 0.016 |
| near-daily / daily vs none | 1.223 | 0.690 | 2.168 | 0.490 | 0.045 | 0.011 |

The table shows hazard ratios (HRs) and 95% confidence intervals (CIs) from the primary multivariable Cox proportional hazards model after multiple imputation, pooled across imputed datasets using Rubin’s rules. “FMI” denotes the fraction of missing information for each coefficient, and “Monte-Carlo error” represents the simulation error of the pooled estimate. Monte-Carlo errors were small relative to the corresponding standard errors, indicating adequate numbers of imputations.

**Table S4** Incident liver disease events, person-years, and incidence rates overall and by CTI quartiles.

| Subgroup | Participants (n) | Events (n) | Person-years (PY) | Incidence rate /1,000 PY (95% CI) |
| --- | --- | --- | --- | --- |
| Overall | 9,302 | 734 | 81,229 | 9.04 (8.39–9.69) |
| Q1 | 2,360 | 153 | 20,609 | 7.42 (6.29–8.55) |
| Q2 | 2,330 | 181 | 20,347 | 8.90 (7.58–10.23) |
| Q3 | 2,311 | 183 | 20,181 | 9.07 (7.72–10.43) |
| Q4 | 2,301 | 217 | 20,093 | 10.80 (9.35–12.25) |

**Table S5** Variance Inflation Factor and tolerance

| Variables | VIF | VIF_CI_low | VIF_CI_high | Tolerance | Tolerance_CI_low | Tolerance_CI_high |
| --- | --- | --- | --- | --- | --- | --- |
| Sex | 2.070 | 1.790 | 2.350 | 0.480 | 0.430 | 0.560 |
| Age | 1.240 | 1.020 | 1.460 | 0.810 | 0.690 | 0.980 |
| Residence | 1.100 | 0.900 | 1.310 | 0.910 | 0.760 | 1.110 |
| Education | 1.380 | 1.150 | 1.610 | 0.720 | 0.620 | 0.870 |
| Smoking | 1.690 | 1.440 | 1.950 | 0.590 | 0.510 | 0.700 |
| Hypertension | 1.080 | 0.880 | 1.280 | 0.920 | 0.780 | 1.140 |
| HeartDisease | 1.050 | 0.850 | 1.250 | 0.950 | 0.800 | 1.180 |
| BMI | 1.000 | 0.810 | 1.200 | 1.000 | 0.830 | 1.240 |
| LDL_C | 1.030 | 0.830 | 1.230 | 0.970 | 0.810 | 1.200 |
| HDL_C | 1.080 | 0.880 | 1.290 | 0.920 | 0.780 | 1.140 |
| Drinking frequency | 1.340 | 1.110 | 1.570 | 0.750 | 0.640 | 0.900 |

**Table S6.** Correlations between CTI and lipid profile parameters

| Variable | CTI | TG | TC | LDL_C | HDL_C |
| --- | --- | --- | --- | --- | --- |
| CTI | 1 | 0.70 (<0.001) | 0.26 (<0.001) | 0.00 (0.657) | -0.48 (<0.001) |
| TG | 0.70 (<0.001) | 1 | 0.31 (<0.001) | -0.16 (<0.001) | -0.44 (<0.001) |
| TC | 0.26 (<0.001) | 0.31 (<0.001) | 1 | 0.80 (<0.001) | 0.19 (<0.001) |
| LDL_C | 0.00 (0.657) | -0.16 (<0.001) | 0.80 (<0.001) | 1 | 0.11 (<0.001) |
| HDL_C | -0.48 (<0.001) | -0.44 (<0.001) | 0.19 (<0.001) | 0.11 (<0.001) | 1 |

Values are Pearson correlation coefficients, with corresponding P values in parentheses.

**Table S7** Schoenfeld residual tests for the Cox model (overall and by sex)

|  | Variables | Chi-square statistic | p |
| --- | --- | --- | --- |
| Overall | CTI | 2.314 | 0.510 |
|  | Sex | 0.737 | 0.391 |
|  | Age | 0.116 | 0.733 |
|  | Residence | 1.843 | 0.175 |
|  | Education | 1.601 | 0.206 |
|  | Smoking | 1.056 | 0.304 |
|  | Hypertension | 0.768 | 0.381 |
|  | HeartDisease | 2.852 | 0.091 |
|  | BMI | 0.234 | 0.628 |
|  | LDL_C | 0.057 | 0.811 |
|  | HDL_C | 0.544 | 0.461 |
|  | Drinking frequency | 2.957 | 0.085 |
|  | GLOBAL | 17.154 | 0.248 |
| Female | CTI | 2.387 | 0.496 |
|  | Age | 0.000 | 0.993 |
|  | Residence | 0.374 | 0.541 |
|  | Education | 0.320 | 0.572 |
|  | Smoking | 0.141 | 0.707 |
|  | Hypertension | 0.060 | 0.806 |
|  | HeartDisease | 3.002 | 0.083 |
|  | BMI | 0.298 | 0.585 |
|  | HDL_C | 0.209 | 0.648 |
|  | LDL_C | 0.610 | 0.435 |
|  | Drinking frequency | 1.230 | 0.267 |
|  | GLOBAL | 9.301 | 0.750 |
| Male | CTI | 0.676 | 0.879 |
|  | Age | 0.152 | 0.697 |
|  | residence | 1.956 | 0.162 |
|  | education | 3.147 | 0.076 |
|  | smoking | 0.285 | 0.594 |
|  | Hypertension | 0.913 | 0.339 |
|  | HeartDisease | 0.515 | 0.473 |
|  | BMI | 0.073 | 0.787 |
|  | HDL_C | 0.304 | 0.581 |
|  | LDL_C | 0.453 | 0.501 |
|  | Drinking frequency | 1.337 | 0.248 |
|  | GLOBAL | 11.550 | 0.565 |

**Table S8**. Standardized 5- and 7-year risks of incident liver disease for CTI Q1 versus Q4

| Time (years) | risk_Q1_pct (%) | risk_Q4_pct (%) | RD_pct (%) |
| --- | --- | --- | --- |
| 5 | 2.960 | 4.030 | 1.071 |
| 7 | 5.431 | 7.356 | 1.925 |

Risk_Q1_pct and Risk_Q4_pct denote the standardized risks for participants in the lowest (Q1) and highest (Q4) CTI quartiles, respectively. RD_pct represents the absolute risk difference (Q4 minus Q1) in percentage points. Standardization was performed using the fully adjusted Cox model with all covariates set to their observed distributions.

**Table S9.** Diagnostic performance from ROC analyses for TG, CRP, CTI, and the composite model

| Variables | AUC | 95% CI | Cutoff-value | Specificity | Sensitivity |
| --- | --- | --- | --- | --- | --- |
| TG | 0.539 | 0.517–0.562 | 172.100 | 0.820 | 0.260 |
| CRP | 0.527 | 0.505–0.549 | 1.445 | 0.630 | 0.420 |
| CTI | 0.542 | 0.520–0.564 | 8.785 | 0.560 | 0.510 |
| Composite variable | 0.604 | 0.583–0.626 | 0.078 | 0.670 | 0.490 |

**Table S10.** Total and direct associations of the CTI with incident liver disease in Cox proportional hazards models

|  | Model 1_total |  | Model 2_total |  | Model 3_total |  | Model 3_direct |  | |
| --- | --- | --- | --- | --- | --- | --- | --- | --- | --- |
| Variable | HR (95% CI) | p | HR (95% CI) | p | HR (95% CI) | p | HR (95% CI) | p |  |
| CTI (per 1-unit) | 1.22 (1.12-1.32) | <0.001 | 1.22 (1.12-1.32) | <0.001 | 1.18 (1.07-1.30) | <0.001 | 1.18 (1.07-1.30) | <0.001 |  |
| CTI quartile |  |  |  |  |  |  |  |  |  |
| Q1 | Ref |  | Ref |  | Ref |  | Ref |  |  |
| Q2 | 1.20 (0.97-1.49) | 0.092 | 1.22 (0.98-1.51) | 0.074 | 1.20 (0.97-1.49) | 0.092 | 1.21 (0.97-1.50) | 0.090 |  |
| Q3 | 1.23 (0.99-1.53) | 0.058 | 1.25 (1.00-1.55) | 0.045 | 1.19 (0.96-1.48) | 0.112 | 1.20 (0.95-1.50) | 0.120 |  |
| Q4 | 1.48 (1.20-1.82) | <0.001 | 1.49 (1.21-1.84) | <0.001 | 1.37 (1.11-1.70) | 0.003 | 1.37 (1.08-1.74) | 0.009 |  |
| P for trend |  | <0.001 |  | <0.001 |  | 0.006 |  | 0.016 |  |

Model 1_total unadjusted.

Model 2_total was additionally adjusted for age, sex, residence, educational level, and smoking status.

Model 3_total was further adjusted for hypertension, heart disease, body mass index (BMI), and drinking frequency.

Model 3_direct was further adjusted for low-density lipoprotein cholesterol (LDL-C), high-density lipoprotein cholesterol (HDL-C).

**Table S11** Sensitivity analysis excluding participants with elevated baseline inflammation (CRP > 10 mg/L): associations between CTI and incident liver disease

| **Characteristic** | **Model** | **1** |  |  | **Model** | **2** |  |  | **Model** | **3** |  |
| --- | --- | --- | --- | --- | --- | --- | --- | --- | --- | --- | --- |
|  | **HR** | **95%CI** | **P** |  | **HR** | **95%CI** | **P** |  | **HR** | **95%CI** | **P** |
| CTI(per 1unit) | 1.27 | 1.17,1.38 | <0.001 |  | 1.27 | 1.17,1.39 | <0.001 |  | 1.25 | 1.13,1.39 | <0.001 |
| CTI quartile |  |  |  |  |  |  |  |  |  |  |  |
| Q1 | Ref |  |  |  | Ref |  |  |  | Ref |  |  |
| Q2 | 1.19 | 0.95,1.48 | 0.126 |  | 1.20 | 0.96,1.50 | 0.109 |  | 1.20 | 0.96,1.50 | 0.118 |
| Q3 | 1.29 | 1.04,1.61 | 0.022 |  | 1.31 | 1.05,1.63 | 0.017 |  | 1.27 | 1.01,1.61 | 0.039 |
| Q4 | 1.52 | 1.23,1.88 | <0.001 |  | 1.54 | 1.24,1.91 | <0.001 |  | 1.44 | 1.12,1.83 | 0.004 |
| P for trend |  |  | <0.001 |  |  |  | <0.001 |  |  |  | 0.004 |

Model 1 unadjusted.

Model 2 was additionally adjusted for sex, age, residence, educational level, and smoking status. Model 3 was further adjusted for hypertension, heart disease, body mass index (BMI), low-density lipoprotein cholesterol (LDL-C), high-density lipoprotein cholesterol (HDL-C), and drinking frequency.

Abbreviations: HR, hazard ratio; CI, confidence interval; CTI, CRP–triglyceride–glucose index; Ref, reference group.

**Table S12.** Sensitivity analysis excluding high-risk alcohol consumers: associations between CTI and incident liver disease

| **Characteristic** | **Model** | **1** |  |  | **Model** | **2** |  |  | **Model** | **3** |  |
| --- | --- | --- | --- | --- | --- | --- | --- | --- | --- | --- | --- |
|  | **HR** | **95%CI** | **P** |  | **HR** | **95%CI** | **P** |  | **HR** | **95%CI** | **P** |
| CTI(per 1unit) | 1.21 | 1.11,1.33 | <0.001 |  | 1.22 | 1.11,1.33 | <0.001 |  | 1.19 | 1.07,1.32 | 0.002 |
| CTI quartile |  |  |  |  |  |  |  |  |  |  |  |
| Q1 | Ref |  |  |  | Ref |  |  |  | Ref |  |  |
| Q2 | 1.15 | 0.91,1.45 | 0.229 |  | 1.17 | 0.92,1.47 | 0.195 |  | 1.15 | 0.91,1.46 | 0.248 |
| Q3 | 1.16 | 0.92,1.46 | 0.220 |  | 1.17 | 0.93,1.48 | 0.186 |  | 1.11 | 0.87,1.43 | 0.399 |
| Q4 | 1.44 | 1.15,1.79 | 0.001 |  | 1.45 | 1.16,1.82 | 0.001 |  | 1.32 | 1.02,1.71 | 0.033 |
| P for trend |  |  | 0.002 |  |  |  | 0.002 |  |  |  | 0.053 |

Model 1 unadjusted.

Model 2 was additionally adjusted for sex, age, residence, educational level, and smoking status. Model 3 was further adjusted for hypertension, heart disease, body mass index (BMI), low-density lipoprotein cholesterol (LDL-C), high-density lipoprotein cholesterol (HDL-C), and drinking frequency.

Abbreviations: HR, hazard ratio; CI, confidence interval; CTI, CRP–triglyceride–glucose index; Ref, reference group.

**Table S13.** Sensitivity analysis excluding early incident liver disease cases at the 2013 wave: associations between CTI and incident liver disease

| **Characteristic** | **Model** | **1** |  |  | **Model** | **2** |  |  | **Model** | **3** |  |
| --- | --- | --- | --- | --- | --- | --- | --- | --- | --- | --- | --- |
|  | **HR** | **95%CI** | **P** |  | **HR** | **95%CI** | **P** |  | **HR** | **95%CI** | **P** |
| CTI(per 1unit) | 1.17 | 1.07,1.28 | <0.001 |  | 1.17 | 1.07,1.28 | <0.001 |  | 1.12 | 1.01,1.25 | 0.035 |
| CTI quartile |  |  |  |  |  |  |  |  |  |  |  |
| Q1 | Ref |  |  |  | Ref |  |  |  | Ref |  |  |
| Q2 | 1.23 | 0.98,1.56 | 0.075 |  | 1.25 | 0.99,1.58 | 0.058 |  | 1.24 | 0.98,1.57 | 0.079 |
| Q3 | 1.20 | 0.95,1.52 | 0.119 |  | 1.22 | 0.97,1.54 | 0.095 |  | 1.16 | 0.91,1.49 | 0.235 |
| Q4 | 1.40 | 1.12,1.75 | 0.003 |  | 1.42 | 1.13,1.78 | 0.002 |  | 1.28 | 0.99,1.66 | 0.062 |
| P for trend |  |  | 0.007 |  |  |  | 0.005 |  |  |  | 0.117 |

Model 1 unadjusted.

Model 2 was additionally adjusted for sex, age, residence, educational level, and smoking status. Model 3 was further adjusted for hypertension, heart disease, body mass index (BMI), low-density lipoprotein cholesterol (LDL-C), high-density lipoprotein cholesterol (HDL-C), and drinking frequency.

Abbreviations: HR, hazard ratio; CI, confidence interval; CTI, CRP–triglyceride–glucose index; Ref, reference group.

**Table S14**. Hazard ratios for incident liver disease from Cox proportional hazards models using age as the time scale

|  | AgeTime_Model 1 |  | AgeTime_Model 2 |  | AgeTime_Model 3 |  |
| --- | --- | --- | --- | --- | --- | --- |
| Variable | HR (95% CI) | p value | HR (95% CI) | p value | HR (95% CI) | p value |
| CTI (per 1-unit) | 1.21 (1.11-1.31) | <0.001 | 1.21 (1.12-1.32) | <0.001 | 1.18 (1.07-1.30) | <0.001 |
| CTI quartile |  |  |  |  |  |  |
| Q1 | Ref |  | Ref |  | Ref |  |
| Q2 | 1.19 (0.96-1.48) | 0.113 | 1.21 (0.97-1.50) | 0.084 | 1.20 (0.97-1.50) | 0.100 |
| Q3 | 1.22 (0.98-1.51) | 0.073 | 1.24 (1.00-1.53) | 0.054 | 1.19 (0.95-1.50) | 0.132 |
| Q4 | 1.45 (1.18-1.78) | <0.001 | 1.48 (1.20-1.82) | <0.001 | 1.36 (1.07-1.72) | 0.012 |
| P for trend |  | <0.001 |  | <0.001 |  | 0.020 |

All models used age as the underlying time scale.

Model 1 unadjusted.

Model 2 was additionally adjusted for sex, age, residence, educational level, and smoking status. Model 3 was further adjusted for hypertension, heart disease, body mass index (BMI), low-density lipoprotein cholesterol (LDL-C), high-density lipoprotein cholesterol (HDL-C), and drinking frequency.

Abbreviations: HR, hazard ratio; CI, confidence interval; CTI, CRP–triglyceride–glucose index; Ref, reference group.

**Table S15.** Subdistribution hazard ratios for incident liver disease from Fine–Gray competing risk models

| Variable | Model 1  sHR (95% CI) | p value | Model 2  sHR (95% CI) | p value | Model 3  sHR (95% CI) | p value |
| --- | --- | --- | --- | --- | --- | --- |
| CTI (per 1-unit) | 1.21 (1.12-1.32) | <0.001 | 1.22 (1.12-1.32) | <0.001 | 1.18 (1.07-1.30) | <0.001 |
| Sex | - | - | 1.02 (0.83-1.25) | 0.860 | 1.10 (0.88-1.37) | 0.390 |
| Age | - | - | 0.99 (0.99-1.00) | 0.160 | 0.99 (0.98-1.00) | 0.009 |
| Residence | - | - | 1.04 (0.89-1.21) | 0.630 | 1.07 (0.92-1.25) | 0.380 |
| Education | - | - | 1.08 (0.97-1.19) | 0.140 | 1.06 (0.95-1.17) | 0.300 |
| Smoking | - | - | 1.10 (0.94-1.28) | 0.220 | 1.06 (0.91-1.23) | 0.440 |
| Hypertension | - | - | - | - | 1.12 (0.96-1.30) | 0.150 |
| HeartDisease | - | - | - | - | 2.07 (1.74-2.46) | <0.001 |
| BMI | - | - | - | - | 1.00 (1.00-1.00) | 0.300 |
| LDL_C | - | - | - | - | 1.00 (1.00-1.00) | 0.590 |
| HDL_C | - | - | - | - | 1.00 (1.00-1.01) | 0.680 |
| Drinking frequency | - | - | - | - | 1.00 (0.93-1.09) | 0.910 |

Model 1 unadjusted.

Model 2 was additionally adjusted for sex, age, residence, educational level, and smoking status. Model 3 was further adjusted for hypertension, heart disease, body mass index (BMI), low-density lipoprotein cholesterol (LDL-C), high-density lipoprotein cholesterol (HDL-C), and drinking frequency.

Death from non–liver disease causes was treated as a competing event.

**Table S16.** Sensitivity analysis using mid-interval event-time assignment (incident LD assigned to the midpoint between two survey waves): associations between CTI and incident liver disease

| **Characteristic** | **Model** | **1** |  |  | **Model** | **2** |  |  | **Model** | **3** |  |
| --- | --- | --- | --- | --- | --- | --- | --- | --- | --- | --- | --- |
|  | **HR** | **95%CI** | **P** |  | **HR** | **95%CI** | **P** |  | **HR** | **95%CI** | **P** |
| CTI(per 1unit) | 1.22 | 1.12,1.32 | <0.001 |  | 1.22 | 1.12,1.32 | <0.001 |  | 1.18 | 1.07,1.30 | <0.001 |
| CTI quartile |  |  |  |  |  |  |  |  |  |  |  |
| Q1 | Ref |  |  |  | Ref |  |  |  | Ref |  |  |
| Q2 | 1.20 | 0.97,1.49 | 0.092 |  | 1.22 | 0.98,1.51 | 0.074 |  | 1.21 | 0.97,1.51 | 0.090 |
| Q3 | 1.23 | 0.99,1.53 | 0.058 |  | 1.25 | 1.00,1.55 | 0.045 |  | 1.20 | 0.95,1.51 | 0.118 |
| Q4 | 1.48 | 1.20,1.82 | <0.001 |  | 1.49 | 1.21,1.84 | <0.001 |  | 1.37 | 1.08,1.74 | 0.009 |
| P for trend |  |  | <0.001 |  |  |  | <0.001 |  |  |  | 0.016 |

Model 1 unadjusted.

Model 2 was additionally adjusted for sex, age, residence, educational level, and smoking status. Model 3 was further adjusted for hypertension, heart disease, body mass index (BMI), low-density lipoprotein cholesterol (LDL-C), high-density lipoprotein cholesterol (HDL-C), and drinking frequency.

Abbreviations: HR, hazard ratio; CI, confidence interval; CTI, CRP–triglyceride–glucose index; Ref, reference group.

**Table S17**. Baseline characteristics according to follow-up status

|  | No follow-up | With follow-up | p | SMD |
| --- | --- | --- | --- | --- |
| Variable | (n = 392) | (n = 9302) |  |  |
| CTI | 9.00 (0.94) | 8.75 (0.84) | <0.001 | 0.278 |
| LDL_C | 114.59 (36.39) | 117.58 (34.95) | 0.098 | 0.084 |
| HDL_C | 50.22 (15.40) | 51.21 (15.31) | 0.21 | 0.064 |
| Sex | 0.53 (0.50) | 0.47 (0.50) | 0.01 | 0.132 |
| Age | 65.24 (11.45) | 58.97 (9.33) | <0.001 | 0.601 |
| Residence | 0.48 (0.50) | 0.63 (0.48) | <0.001 | 0.308 |
| Education | 0.89 (0.81) | 1.03 (0.80) | 0.001 | 0.174 |
| Smoking | 0.59 (0.72) | 0.47 (0.65) | <0.001 | 0.172 |
| Hypertension | 0.58 (0.49) | 0.45 (0.50) | <0.001 | 0.275 |
| HeartDisease | 0.14 (0.35) | 0.13 (0.34) | 0.727 | 0.018 |
| BMI | 23.06 (4.45) | 24.08 (27.22) | 0.458 | 0.052 |
| Drinking frequency | 0.63 (1.08) | 0.67 (1.08) | 0.517 | 0.033 |

Values are presented as mean (standard deviation) for continuous variables. Participants were categorized into those without follow-up (n = 392) and those with follow-up (n = 9,302). P values compare groups using Student’s t test for approximately normally distributed continuous variables and the χ² test for categorical variables, as appropriate. Standardized mean differences (SMDs) quantify the magnitude of between-group differences and are calculated as the difference in group means divided by the pooled standard deviation. An absolute SMD ≥0.10 was considered to indicate a meaningful imbalance.

**Table S18.** Inverse-probability-of-censoring-weighted Cox regression for incident liver disease

| Variable | HR | CI_low | CI_high | p value |
| --- | --- | --- | --- | --- |
| CTI | 1.180 | 1.071 | 1.300 | 0.001 |
| Sex | 1.101 | 0.882 | 1.374 | 0.394 |
| Age | 0.988 | 0.980 | 0.996 | 0.005 |
| BMI | 1.000 | 0.999 | 1.000 | 0.325 |
| Smoking | 1.063 | 0.912 | 1.239 | 0.432 |
| Hypertension | 1.120 | 0.961 | 1.306 | 0.147 |
| HeartDisease | 2.092 | 1.752 | 2.499 | <0.001 |
| LDL_C | 0.999 | 0.997 | 1.002 | 0.599 |
| Residence | 1.075 | 0.922 | 1.254 | 0.354 |
| Education | 1.057 | 0.953 | 1.172 | 0.292 |
| HDL_C | 1.001 | 0.995 | 1.007 | 0.706 |
| Drinking frequency | 1.005 | 0.928 | 1.088 | 0.901 |

Hazard ratios (HRs) and 95% confidence intervals (CIs) are from a Cox proportional hazards model fitted with inverse-probability-of-censoring weights (IPCW) to account for loss to follow-up. Weights were derived from a logistic regression model predicting having follow-up versus being lost to follow-up based on baseline covariates, and were stabilized before being applied to the outcome model. CTI and all listed covariates were included in the weighted Cox model.

**Table S19.** Sensitivity analysis using a more stringent endpoint definition (requiring both self-reported physician diagnosis and liver-disease medication use): associations between CTI and incident liver disease

| **Characteristic** | **Model** | **1** |  |  | **Model** | **2** |  |  | **Model** | **3** |  |
| --- | --- | --- | --- | --- | --- | --- | --- | --- | --- | --- | --- |
|  | **HR** | **95%CI** | **P** |  | **HR** | **95%CI** | **P** |  | **HR** | **95%CI** | **P** |
| CTI(per 1unit) | 1.20 | 1.07,1.35 | 0.002 |  | 1.22 | 1.08,1.37 | 0.001 |  | 1.21 | 1.05,1.39 | 0.007 |
| CTI quartile |  |  |  |  |  |  |  |  |  |  |  |
| Q1 | Ref |  |  |  | Ref |  |  |  | Ref |  |  |
| Q2 | 1.23 | 0.91,1.67 | 0.182 |  | 1.25 | 0.92,1.70 | 0.148 |  | 1.26 | 0.92,1.72 | 0.145 |
| Q3 | 1.20 | 0.88,1.64 | 0.239 |  | 1.24 | 0.91,1.69 | 0.172 |  | 1.22 | 0.88,1.69 | 0.237 |
| Q4 | 1.37 | 1.20,1.85 | 0.037 |  | 1.43 | 1.06,1.93 | 0.021 |  | 1.35 | 0.96,1.91 | 0.086 |
| P for trend |  |  | 0.055 |  |  |  | 0.030 |  |  |  | 0.129 |

Model 1 unadjusted.

Model 2 was additionally adjusted for sex, age, residence, educational level, and smoking status. Model 3 was further adjusted for hypertension, heart disease, body mass index (BMI), low-density lipoprotein cholesterol (LDL-C), high-density lipoprotein cholesterol (HDL-C), and drinking frequency.

Abbreviations: HR, hazard ratio; CI, confidence interval; CTI, CRP–triglyceride–glucose index; Ref, reference group.

**Table S20**. Baseline characteristics of incident liver disease cases according to use of liver-related medication

|  | No medication | Medication | p | SMD |
| --- | --- | --- | --- | --- |
| Variable | (n=359) | (n=375) |  |  |
| CTI | 8.86 (0.88) | 8.92 (0.94) | 0.359 | 0.068 |
| LDL_C | 116.97 (36.31) | 116.48 (40.43) | 0.862 | 0.013 |
| HDL_C | 49.51 (15.36) | 50.41 (16.43) | 0.445 | 0.057 |
| Sex | 0.51 (0.50) | 0.48 (0.50) | 0.379 | 0.065 |
| Age | 58.18 (8.03) | 58.70 (8.62) | 0.403 | 0.062 |
| Residence | 0.57 (0.50) | 0.67 (0.47) | 0.005 | 0.209 |
| Education | 1.17 (0.79) | 1.03 (0.77) | 0.017 | 0.177 |
| Smoking | 0.52 (0.71) | 0.53 (0.71) | 0.849 | 0.014 |
| Hypertension | 0.49 (0.50) | 0.51 (0.50) | 0.657 | 0.033 |
| HeartDisease | 0.20 (0.40) | 0.27 (0.45) | 0.029 | 0.162 |
| BMI | 24.61 (4.01) | 23.77 (4.24) | 0.006 | 0.205 |
| Drinking frequency | 0.75 (1.13) | 0.63 (1.07) | 0.168 | 0.102 |

Standardized mean differences (SMDs) quantify the magnitude of between-group differences and are calculated as the difference in group means divided by the pooled standard deviation. An absolute SMD ≥0.10 was considered to indicate a meaningful imbalance.

**Table S21**.Sensitivity analysis restricted to participants with repeated measurements of CTI components (repeat-measurement subset): associations between CTI and incident liver disease

| **Characteristic** | **Model** | **1** |  |  | **Model** | **2** |  |  | **Model** | **3** |  |
| --- | --- | --- | --- | --- | --- | --- | --- | --- | --- | --- | --- |
|  | **HR** | **95%CI** | **P** |  | **HR** | **95%CI** | **P** |  | **HR** | **95%CI** | **P** |
| CTI(per 1unit) | 1.25 | 1.14,1.38 | <0.001 |  | 1.26 | 1.14,1.38 | <0.001 |  | 1.25 | 1.11,1.40 | <0.001 |
| CTI quartile |  |  |  |  |  |  |  |  |  |  |  |
| Q1 | Ref |  |  |  | Ref |  |  |  | Ref |  |  |
| Q2 | 1.13 | 0.87,1.46 | 0.368 |  | 1.13 | 0.87,1.47 | 0.349 |  | 1.14 | 0.87,1.48 | 0.337 |
| Q3 | 1.27 | 0.99,1.64 | 0.060 |  | 1.27 | 0.99,1.64 | 0.062 |  | 1.24 | 0.95,1.63 | 0.110 |
| Q4 | 1.53 | 1.20,1.96 | <0.001 |  | 1.54 | 1.21,1.97 | <0.001 |  | 1.47 | 1.11,1.94 | 0.007 |
| P for trend |  |  | <0.001 |  |  |  | <0.001 |  |  |  | 0.006 |

Model 1 unadjusted.

Model 2 was additionally adjusted for sex, age, residence, educational level, and smoking status. Model 3 was further adjusted for hypertension, heart disease, body mass index (BMI), low-density lipoprotein cholesterol (LDL-C), high-density lipoprotein cholesterol (HDL-C), and drinking frequency.

Abbreviations: HR, hazard ratio; CI, confidence interval; CTI, CRP–triglyceride–glucose index; Ref, reference group.


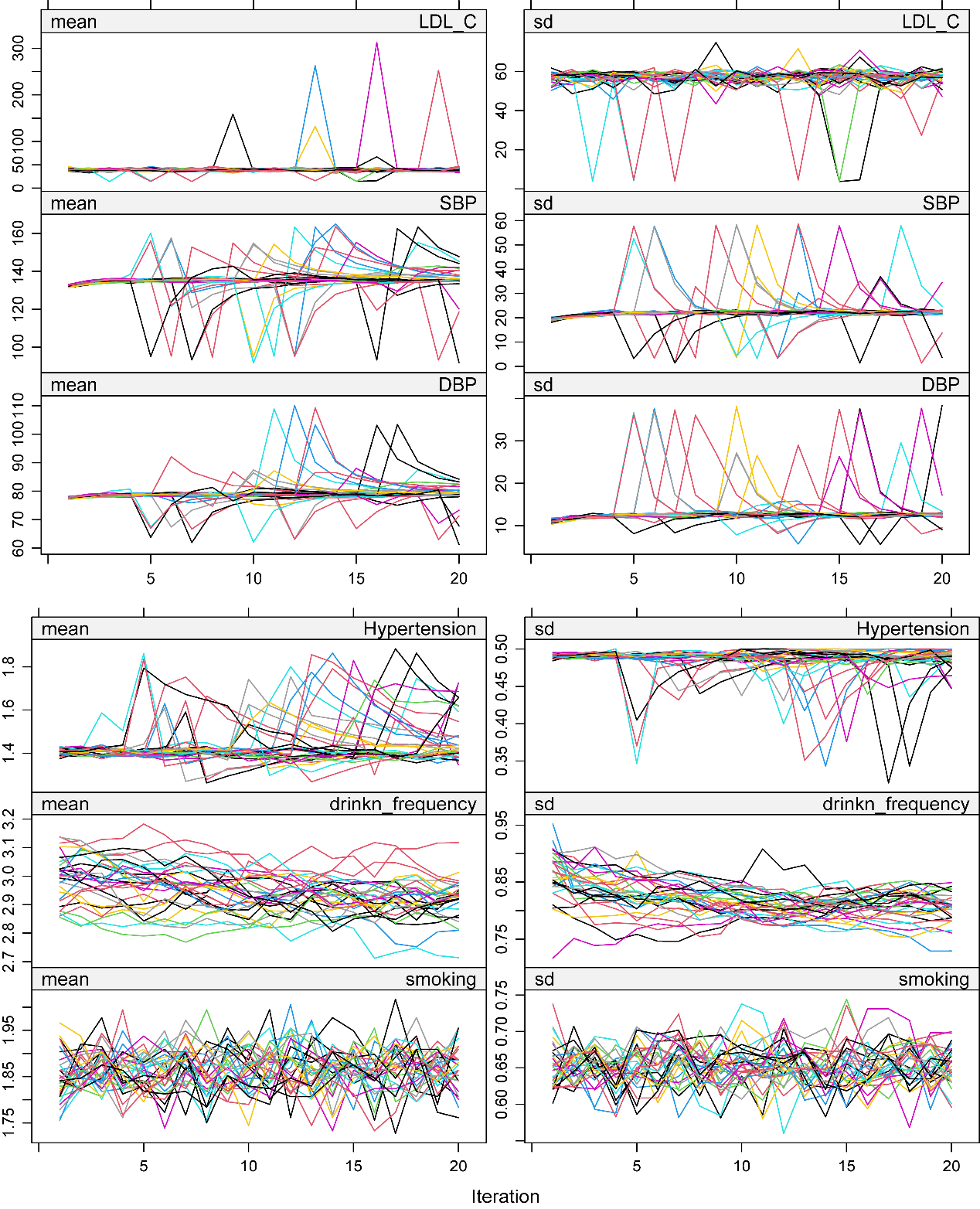


**Figure S1.** Trace plots of the mean and standard deviation for selected continuous and categorical variables across 20 iterations of the multiple imputation algorithm. Each colored line represents a single imputed dataset.


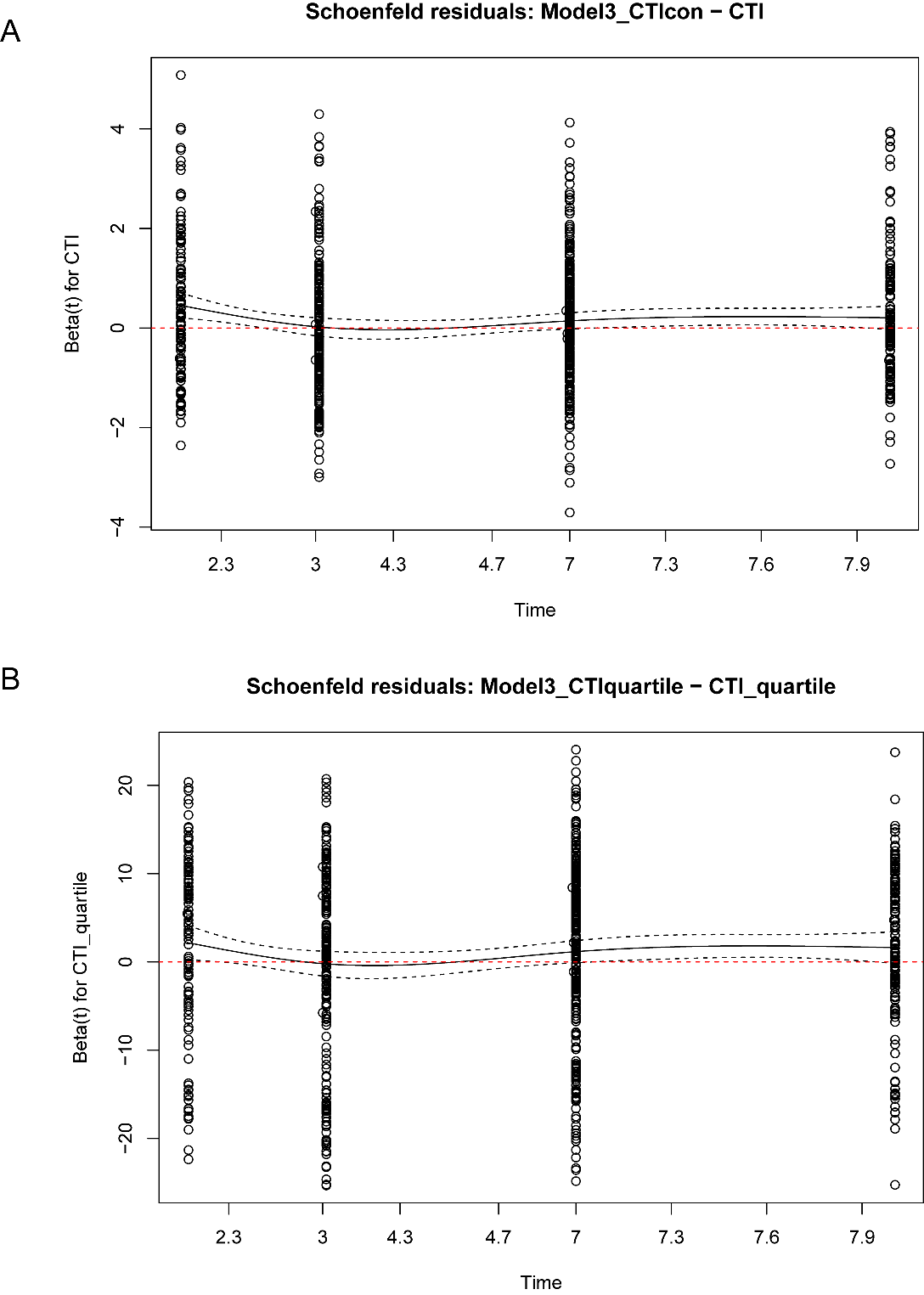


**Figure S2.** Schoenfeld residual plots were used to evaluate the proportional hazards assumption for the fully adjusted Cox models (Model 3). (A) CTI modeled as a continuous variable. (B) CTI modeled in quartiles (entered as a categorical factor). Points represent scaled Schoenfeld residuals at event times; the solid curve shows the smoothed time-varying coefficient estimate, and dashed curves indicate the approximate 95% confidence bands. The horizontal reference line at 0 denotes no time-varying effect; absence of a systematic departure from 0 suggests no meaningful violation of the proportional hazards assumption.


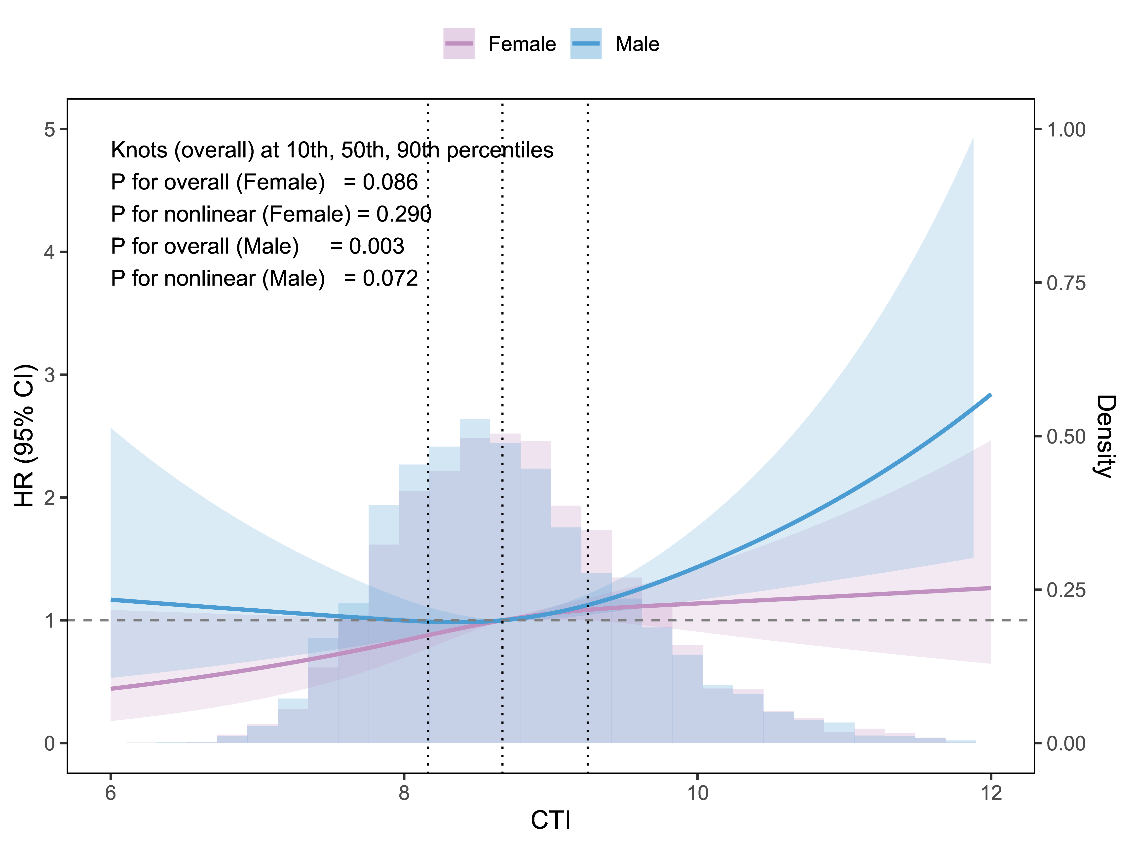


**Figure S3.** Sex-stratified restricted cubic spline dose–response association between CTI and incident liver disease. Restricted cubic spline (RCS) models were fitted separately in females and males to examine the dose–response relationship between continuous CTI and incident liver disease. Knots were placed at the 10th, 50th, and 90th percentiles of CTI. Hazard ratios (HRs) are shown with 95% confidence bands and are referenced to HR = 1 at the median CTI. Shaded histograms display the sex-specific distribution (density) of CTI. Dotted vertical lines indicate knot locations. P values for the overall association and for nonlinearity (Wald tests) are annotated for each sex.


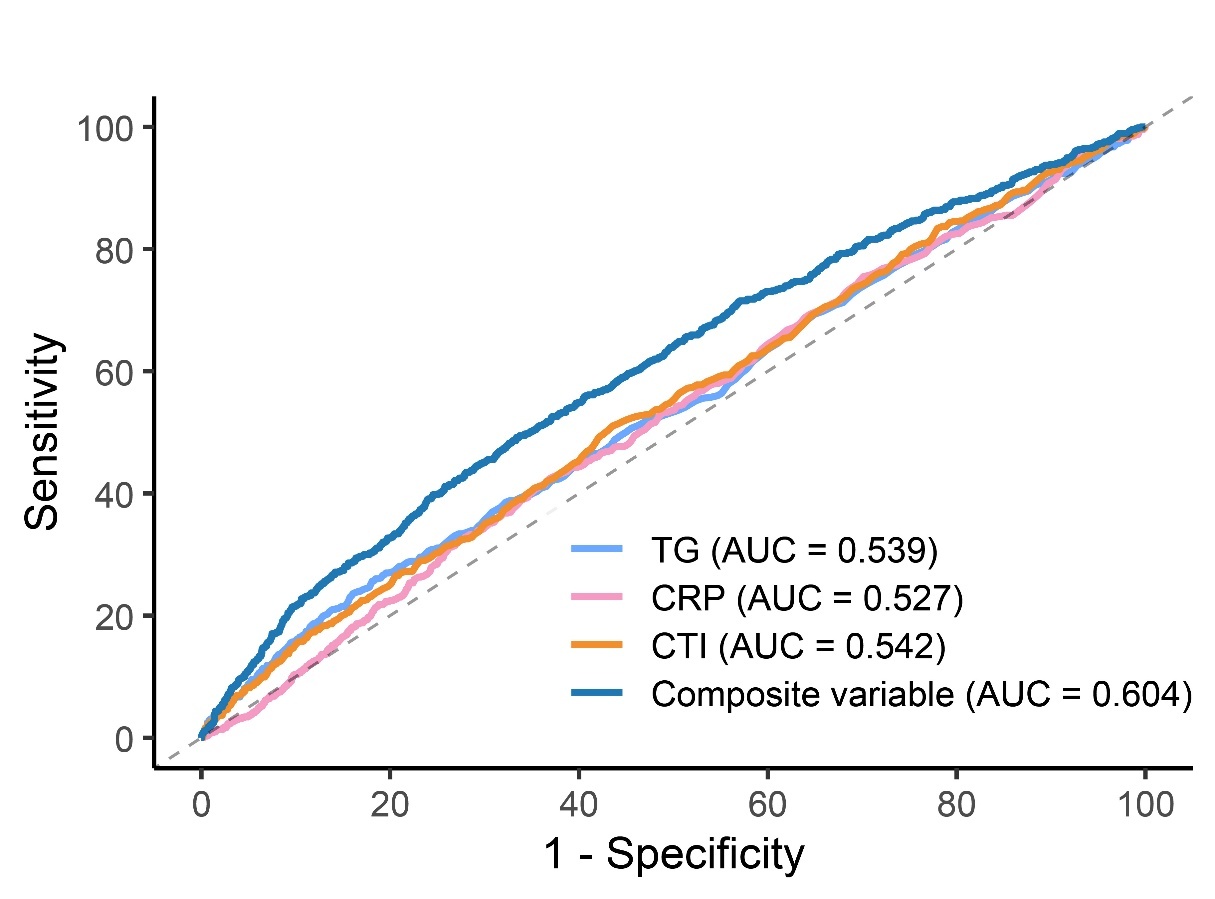


**Figure S4.** ROC curves comparing TG, CRP, CTI, and the composite model for liver-related outcomes. The composite model includes CTI, Age, Gender, Residence, Marital status, Education level, Smoking status, Drinking status, Hypertension, Heart disease, BMI, and LDL-C.
